# Development of a V5-tag–directed nanobody and its implementation as an intracellular biosensor of GPCR signaling

**DOI:** 10.1016/j.jbc.2023.105107

**Published:** 2023-07-28

**Authors:** Manel Zeghal, Kevin Matte, Angelica Venes, Shivani Patel, Geneviève Laroche, Sabina Sarvan, Monika Joshi, Jean-Christophe Rain, Jean-François Couture, Patrick M. Giguère

**Affiliations:** 1 Department of Biochemistry, Microbiology and Immunology, Faculty of Medicine, University of Ottawa, Ottawa, Ontario, Canada; 2 Ottawa Institute of Systems Biology, University of Ottawa, Ottawa, Ontario, Canada; 3 Hybrigenics Services, Évry-Courcouronnes, France; 4 Brain and Mind Research Institute, University of Ottawa, Ottawa, Ontario, Canada

**Keywords:** protein-protein interaction, single-domain antibody (sdAb, nanobody), crystal structure, G protein-coupled receptor (GPCR), bioluminescence resonance energy transfer (BRET), protease-dependent reporter assay (Tango), nanoluciferase binary technology (NanoBiT)

## Abstract

Protein–protein interactions (PPIs) form the foundation of any cell signaling network. Considering that PPIs are highly dynamic processes, cellular assays are often essential for their study because they closely mimic the biological complexities of cellular environments. However, incongruity may be observed across different PPI assays when investigating a protein partner of interest; these discrepancies can be partially attributed to the fusion of different large functional moieties, such as fluorescent proteins or enzymes, which can yield disparate perturbations to the protein’s stability, subcellular localization, and interaction partners depending on the given cellular assay. Owing to their smaller size, epitope tags may exhibit a diminished susceptibility to instigate such perturbations. However, while they have been widely used for detecting or manipulating proteins *in vitro*, epitope tags lack the *in vivo* traceability and functionality needed for intracellular biosensors. Herein, we develop NbV5, an intracellular nanobody binding the V5-tag, which is suitable for use in cellular assays commonly used to study PPIs such as BRET, NanoBiT, and Tango. The NbV5:V5 tag system has been applied to interrogate G protein–coupled receptor signaling, specifically by replacing larger functional moieties attached to the protein interactors, such as fluorescent or luminescent proteins (∼30 kDa), by the significantly smaller V5-tag peptide (1.4 kDa), and for microscopy imaging which is successfully detected by NbV5-based biosensors. Therefore, the NbV5:V5 tag system presents itself as a versatile tool for live-cell imaging and a befitting adaptation to existing cellular assays dedicated to probing PPIs.

Nestled at the heart of the study of molecular biology, protein–protein interaction (PPI) assays stand as heralded experimental techniques used to investigate the interactions between two or more proteins, often informing drug discovery and development, such as for G protein–coupled receptor (GPCR) signaling (1, 2). Cell-based assays also find particular relevance in unraveling PPIs from a physiological context, shedding light on their roles in biological processes, their implications within cellular environments, and the consequences of their dysregulation in disease (3). Considering the varying strengths and limitations inherent to each approach, the selection of an appropriate PPI assay is contingent upon the specific research question being addressed (4). Earlier experimental methodologies, such as pull-down and immunoprecipitation techniques, were commonly employed to detect or isolate protein complexes. However, these approaches exhibit disruptive tendencies towards larger complexes, particularly those involving membrane-spanning proteins (5). A pivotal subset of cell-based assays comes in the form of biosensor-based techniques, which offer indispensable insights into PPIs within the realm of living cells. By utilizing fluorescent/luminescent molecules or functional moieties to label two proteins of interest, the biosensor-based technique enables monitoring their interaction within a cellular milieu. Moreover, these cellular biosensors can measure both high- and low-affinity interactions and transient and stable complexes using endpoint or live assay (6, 7). With respect to GPCR signaling, the most common cellular systems employing such biosensors include the protease-dependent reporter assays such as the Tobacco Etch protease-dependent assay (Tango), bioluminescence resonance energy transfer (BRET), and Nanoluciferase binary technology (NanoBiT) (8). Despite the value of these biosensor-based cellular techniques, the imbroglio of the protein interactome can complicate the execution of its study, and investigations of PPIs at a scaled-up level have been limited thus far. Moreover, PPI assays, such as resonance energy transfer or binary complementation, require cloning of each component in all possible and permissive orientations, specifically at the C terminus or N terminus of the protein, which could translate into over twelve different combinations to be tested (9). Finally, while approaches such as Tango, BRET, and NanoBiT have been designed to provide an understanding of different PPIs, all involve the fusion of large, functionalized tags (tobacco etch virus (TEV), GFP2, Large BiT (LgBiT), respectively) to the proteins of interest (10).

Considering the aforementioned limitations, the adoption of short peptide or epitope tags offers a reasonable possibility to probe PPIs. Less than 20 residues in length, epitope tags (*e.g.*, HA, Flag, Myc, and V5) are commonplace in protein purification and localization/detection such as microscopy but less so for monitoring dynamic interactions (11). However, although fluorophores (*e.g.*, GFP, mCherry) and other polypeptides (*e.g.*, MBP, GST) are popularly used for probing recombinant proteins in a cellular context, including live cell imaging, reporter assays, and resonance energy transfer, the smaller size of epitope tags could be preferable for these purposes, as the fusion of bulkier fluorophores and polypeptides could interfere with the native behavior and functions of the protein of interest, especially interactions that are particularly proximity- or location-dependent (12). Besides avoiding cross-reactions within the native proteome, epitope tags obviate the need for custom antibody generation for every protein of interest and give rise to the possibility of multiplexing orthogonal peptide tags within the same experiment (11, 13).

Given the indispensability of these peptides, antibodies have continually been developed to recognize existing and newly developed epitope tags. Although conventional antibodies have proven to be robust workhorses in various cell biology research fields, their large size and complex architecture preclude them from functioning within living cells (14, 15). These limitations spurred advances in engineering novel recombinant antibody constructs, termed intracellular antibodies or intrabodies. Although their biophysical properties are unlike the conventional monoclonal antibodies, intrabodies retain the capacities for recognition and/or neutralization with similarly high specificities and affinities, with the added conferred ability to bind specific targets expressed within the same living cell that houses it, typically within the cytoplasm of eukaryotic cells (16). Despite intense efforts, minimal antigen-binding fragments such as single-chain variable fragments or F(ab)-type fragments remain highly unstable in a cellular environment and have limited activity in an intracellular milieu (17, 18, 19). The challenges for intrabodies to be expressed in their functional forms are partly due to their aggregation propensity and the reducing environment of the cytoplasm, which prevents intradomain disulfide bond formation (20, 21).

Of greater promise are single-domain antibodies, also referred to as variable domain of heavy-chain antibodies (VHHs) or nanobodies (Nbs), which are the smallest antibody derivatives (12–15 kDa). Although originally derived from members of the Camelidae and *Chondrichthyes* family, human-derived single-domain antibodies have also been engineered from conventional human IgGs (22). Epitope-specific Nbs can be selected by various approaches, including display-based panning or synthetic libraries generated *in vitro* using predesigned scaffolds that undergo complementarity-determining region (CDR) codon randomization (23). Moreover, retrieved Nbs can be affinity-matured to further improve upon the original pharmacological features (24). Structurally, these noncanonical antibodies are more compact, consisting exclusively of shortened heavy chains with three interspersed CDRs that form their antigen-binding domain. Devoid of the hydrophobic interface between heavy and light chains and stabilized by only a single internal disulfide bond, Nbs rival conventional IgGs given their low immunogenicity, enhanced solubility, and stability, all while retaining high binding affinities towards their targets and thereby are more successfully expressed in the cytosol of mammalian cells (25). In terms of binding performance, Nbs have an enhanced ability to target sterically hindered and/or concave epitopes given their slightly convex-shaped paratope (26). As such, the physicochemical and pharmacological properties of Nbs offer a multitude of possibilities for live-cell applications, such as elucidating signaling pathways and PPIs in live cells, targeting proteins or protein complexes formerly inaccessible to biologics, fusing to other functional effectors such as proteases and fluorophores, and visualizing and localizing proteins of interest using conventional and advanced microscopy (27). Moreover, Nbs targeting intracellular targets are plentiful, the majority of which target various cytosolic proteins and more recently, short peptide or epitope tags (28, 29, 30, 31, 32, 33, 34, 35, 36, 37, 38, 39, 40, 41). However, Nbs recognizing these small peptide fragments are still scarce, in part due to conformational difficulties of the cleft-like paratope of Nbs for recognizing linear peptides and the needed strength of Nb–peptide tag interaction; based on existing literature, less than 10 peptide-targeted Nbs have been identified, specifically against the EPEA (42), SPOT (43, 44), 6E (45), Moon (46, 47), Myc (48), BC2 (43, 49), and ALFA tags (50, 51). Only two of these antibody-peptide pairs retained binding in cells, the Moon and ALFA tag, allowing for the possibility of multiplexing (52).

Therefore, the paucity of tag-targeted intrabodies is a bottleneck for intracellular interrogations, especially given the pre-existing arrayed or pooled tag-encoded complementary DNA (cDNA) and ORF libraries for overexpression studies. For example, while there are comprehensive human genome-wide and fully annotated V5-tagged libraries developed from the ORFeome project (53) and the *Drosophila* genome-wide V5-tagged library (54), these open resources are mainly limited to phenotypic screenings for living cellular assay or microscopy for *in vitro* assay on fixed tissues. As for the peptide itself, the V5 tag is a universal epitope that has been extensively used since its introduction roughly 30 years ago (55) and has desirable characteristics suitable for intracellular use. More specifically, the V5 tag is of small size (1.42 kDa), possesses an equal number of positively and negatively charged residues exhibiting a pI of 5.85 and no net charge at physiological pH (56, 57), is of prokaryotic origin which circumvents possible background signals when expressed in mammalian and insect cell host, as well as being resistant to cleavage by their endogenous proteases (51). Thus, the development of intracellular Nb recognizing the small peptide V5-tag and its functionalization would be highly amenable to cellular studies of a broader nature using the genome-wide V5-tagged ORF library, as well as more directed investigations of gene function and genetic rescues and protein–protein interactions such as dynamic receptor-protein interfaces, with minimal interference.

To this end, we introduce the NbV5, a novel Nb that interacts with the V5-tag, serving as the foundation of our multipurpose intracellular nanobody-based biosensors. Our work demonstrates that the NbV5:V5 tag system is suitable for monitoring dynamic PPIs in various adapted cellular assays, specifically in the context of GPCR signaling, and for microscopic imaging techniques.

## Results

### Generation of a nanobody directed against the V5-tag

**Figure 1:**
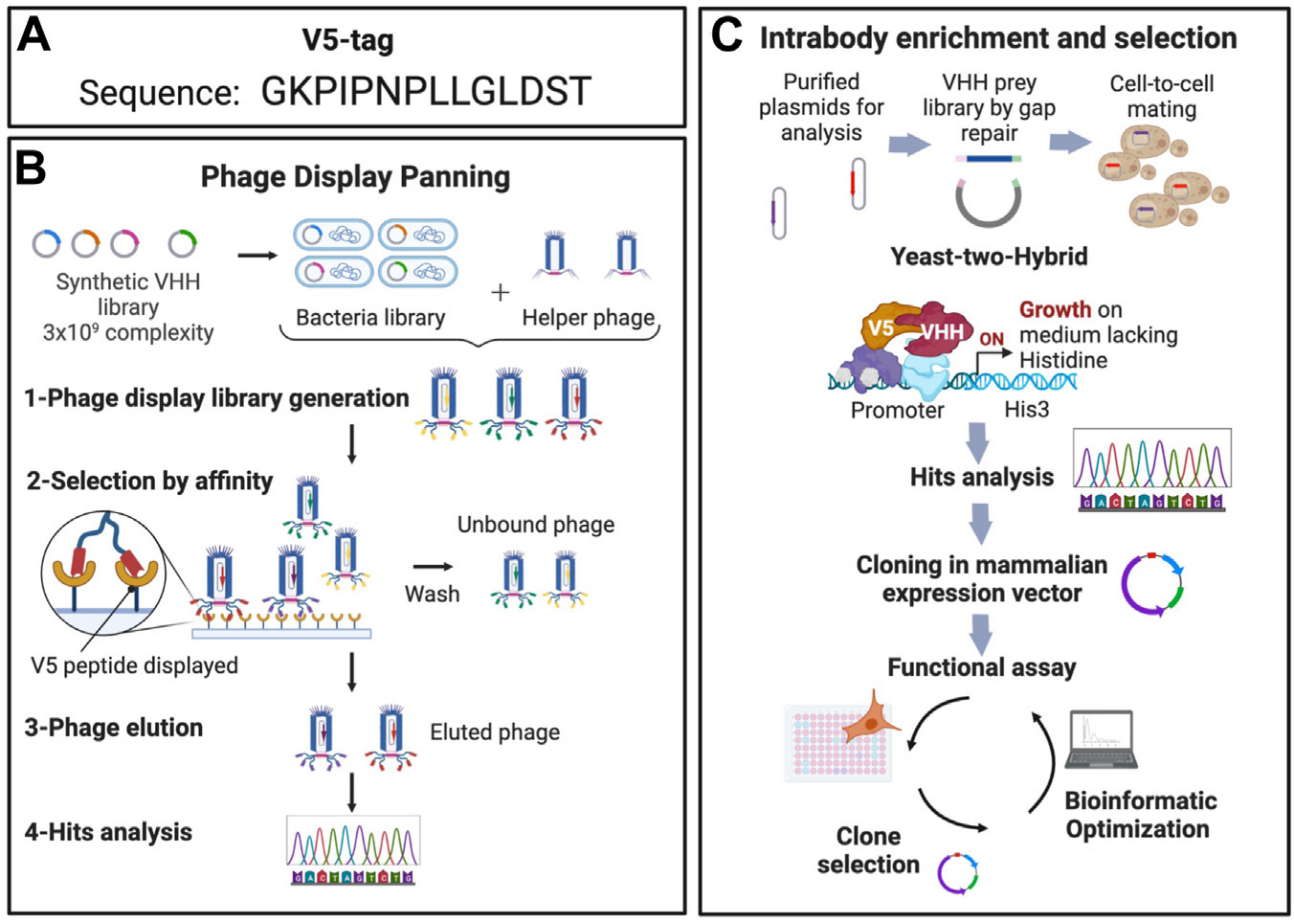
**Overview of the selection of a synthetic nanobody interacting with the V5-tag.***A*, sequence of the V5-tag. *B*, schematic of the phage-display panning for the enrichment of nanobodies interacting with the V5-peptide tag. *C*, schematic of the yeast two-hybrid screening (Y2H) for the enrichment of nanobodies interacting with the V5-peptide tag in an intracellular environment (intrabody).

The first generation of selective Nbs recognizing the V5-tag was identified using the Hybribody approach, a two-pronged screening strategy involving synthetic VHHs phage display and yeast two-hybrid (Y2H). Peptide phage display was first performed against a purified HaloTag protein harboring a duplicate copy of the V5-tag to select for a synthetic VHH against the V5-tag (sequence in Fig. 1*A*). Following the first round of the phage display screening process, the VHHs demonstrating an interaction for the V5-tag were subsequently cloned into a yeast prey vector to execute the second Y2H (58, 59), yielding 52 distinct VHHs with redundancies ranging from 1 to 37. Among this repertoire of VHHs, the top 20 clones that exhibited the highest degrees of redundancy were selected and subsequently cloned into mammalian expression vector fused at the C terminus with the TEV219. This integrated approach and the utilization of the highly diverse humanized library NaLi-H1 (60) promoted the enrichment of stable and soluble intracellular-targeting Nbs; the overall process is illustrated in Figure 1, *B* and *C*.

**Figure 2:**
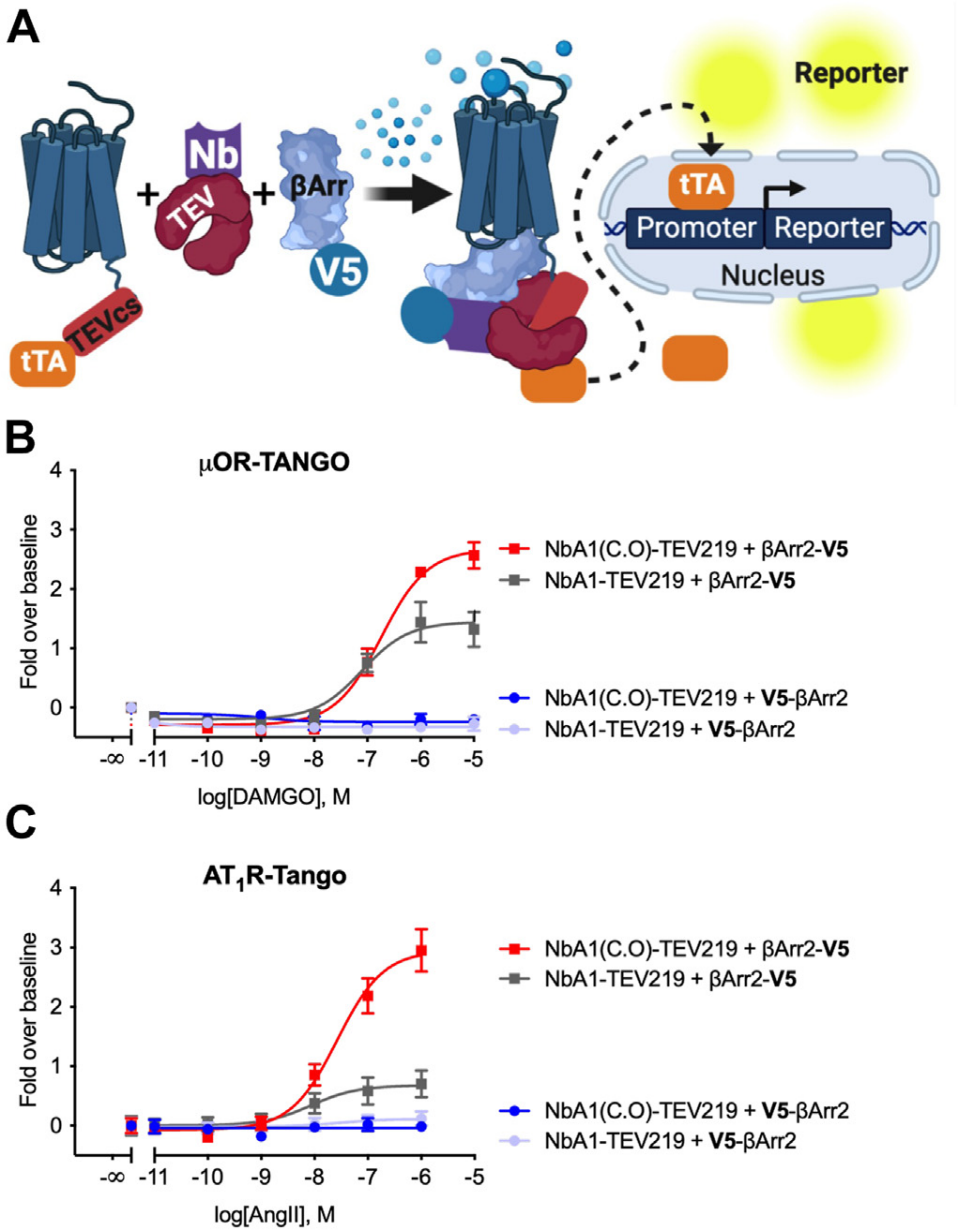
**The first generation of the anti-V5 nanobody (NbA1) only recognizes the C-terminal–positioned V5-tag.***A*, schematic of the protease-dependent cell-based assay (TANGO) used to assay the anti-V5 nanobodies. The original NbA1 clone selected from the Y2H screening was fused with the TEV219 protease and cloned into a eukaryotic expression vector. The initial assessment revealed that the nanobody was not well expressed in HEK293 and codon optimization (NbA1(C.O)) increased its expression and consequently its functional activity. The μ-OR-TANGO (*B*) and AT_1_R-TANGO (*C*) were cotransfected with β-arrestin2 carrying a C- or N-terminal V5-peptide tag along with either the NbA1-TEV219 or NbA1(C.O)-TEV219 fusion protein the receptor stimulated with dose-response agonists, DAMGO for μ-OR and angiotensin II (AngII) for AT_1_R. The original NbA1 clone only recognizes the C-terminally tagged β-arrestin2 (β-arrestin2-V5). Dose-response curves were built using XY analysis for nonlinear regression curve and the 3-parameters dose-response stimulation function from GraphPad Prism. Baseline corrected curves were built using the “Remove baseline and column math” function (Value-Baseline/Baseline). Wells in the absence of ligand were used as the baseline for each condition. All error bars represent SD of three or four technical replicates. Data presented are representative of three biological replicates. μ-OR, mu-opioid receptor; AT1R, angiotensin II receptor type 1; HEK293, human embryonic kidney 293; TEV, tobacco etch virus; Y2H, yeast two-hybrid.

To interrogate their binding capabilities and specificities, the potential VHHs were subsequently evaluated using the Tango-based assay, specifically by using the mu-opioid receptor (μ-OR) and angiotensin II receptor type 1 (AT1R) and independently testing each clone using increasing concentrations of the μ-OR agonist DAMGO and AT1R agonist angiotensin II. To maximize the detection of β-arrestin recruitment at these receptors, two variants of tagged-β-arrestin-2 were utilized, either carrying an N- or C-terminal V5-tag (V5-β-arrestin-2 and β-arrestin-2-V5, respectively). The overall schematic of the nanobody-based Tango assay is depicted in Figure 2*A*. Based on these preliminary findings, a particular clone, designated as NbA1, exhibited a robust response with β-arrestin-2-V5 while displaying a barely detectable response with N-terminally tagged V5-β-arrestin-2. Furthermore, codon optimization for human expression enhanced the original nanobody's expression level threefold and thus was selected for further experiments in human cells (Fig. 2, *B* and *C*).

### Crystal structure of the NbA1

**Figure 3:**
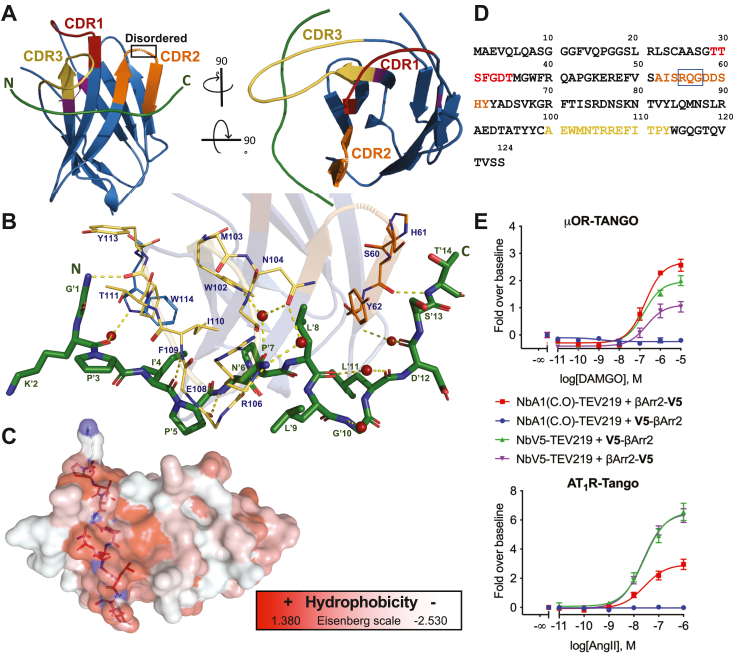
**Structure of the NbA1 bound to the V5 peptide.***A*, overview of the NbA1:V5 peptide complex shown as cartoon representation. NbA1 is colored in *blue* with CDRs 1 to 3 colored in *red*, *orange*, and *yellow*, respectively. Three residues from the CDR2 were not resolved in the refined structure. *B*, close-up view of the polar interactions within the complex including water-bridged interaction. The V5-peptide (*green*) is shown as stick representation with the N terminal on the left. Interacting residues from the paratope are labeled in *blue*, while the peptide labels are *green* and marked with a prime symbol. H-bonds are shown as *yellow dashed lines* and water molecules as *red spheres*. *C*, the NbA1:V5 peptide complex shown as surface representation and colored using the color_h script based on the Eisenberg hydrophobicity scale ^64^. *D*, sequence of NbA1 with CDRs 1 to 3 colored in *red*, *orange*, and *yellow*, respectively. The square highlights the RQG tripeptide that is disordered in the CDR2 loop. *E*, the μ-OR-TANGO and AT_1_R-TANGO were cotransfected with β-arrestin2 carrying a C- or N-terminal V5-peptide tag along with either the NbV5-TEV219 or codon-optimized NbA1(C.O)-TEV219 fusion protein the receptor stimulated with dose-response agonists, DAMGO for μ-OR and angiotensin II (AngII) for AT_1_R. The NbV5 recognizes the N- or C-terminally V5-tagged β-arrestin2 with similar logistic parameters (potency and efficacy), while the original NbA1 clone only recognizes the C-terminally tagged β-arrestin2 (β-arrestin2-V5). Dose-response curves were built using XY analysis for nonlinear regression curve and the 3-parameters dose-response stimulation function from GraphPad Prism. Baseline correction was performed using the “Remove baseline and column math” function (Value-Baseline/Baseline). Wells in the absence of ligand were used as the baseline for each condition. All error bars represent SD of three or four technical replicates. Data presented are representative of three biological replicates. μ-OR, mu-opioid receptor; AT1R, angiotensin II receptor type 1; CDR, complementarity-determining region; TEV, tobacco etch virus.

To address the suboptimal activity of NbA1 toward the N-terminally tagged β-arrestin-2, a computer-aided affinity maturation strategy was opted for. Towards this aim, we initially solved the V5-bound structure of NbA1 at a resolution of 2 Å (Table 1). Structural analysis revealed that NbA1 adopts a typical V-shaped IgG fold connected by a disulfide bond, while the V5 peptide lacks any secondary structures (Fig. 3*A*) (61). The paratope responsible for V5 peptide interaction is primarily mediated by the CDR2 and CDR3 regions, a common feature of Nbs (62). Nbs compensate for their reduced number of CDRs compared to conventional antibodies through distinctive CDR structures and antigen-binding modes; for example, many Nbs possess elongated CDR3 loops to provide a larger antigen interaction surface (43), as observed in NbV5 (Fig. 3*A*). The V5 peptide makes multiple polar and hydrophobic contacts with NbA1 and is oriented at 90 degrees to the central axis of the nanobody. Out of the 14 residues of the tag, 11 directly interact with NbA1 (Fig. 3*B*). The lateral chains of two residues, Lys 2 and Ile 4, point outward but their backbones make water-mediated hydrogen bonds with NbA1. As shown in Figure 3*C*, the interaction is largely mediated by hydrophobic residues, suggesting a strong contribution of entropic forces by the Nb:V5 interfaces (63). Numerous reports have documented the greater paratope diversity of Nbs than the classical Ab VH domain (63), which was exemplified in this study by the distinct recognition mode of NbA1:V5 compared to the NbALFA:ALFA-tag interaction, which involves contacts with all three CDRs and is oriented parallel to the central axis (Fig. S1) of the nanobody. Finally, three residues (Gln57, Gly58, Asp59) within the CDR2 loop are disordered and were unresolved (Figs. 3*A* and S1). The primary coding sequence of NbA1 is shown in Figure 3*D*.

**Table 1:** Data collection and refinement statistics for NbA1 in complex with the V5 peptide

	NbA1:V5
**Data collection**	
Space group	P1
Cell dimensions	
*a*, *b*, *c* (Å)	45.4, 45.4, 70.6
α,β,γ (°)	90.0, 90.0, 97.9
Resolution (Å)	24.59-2.01 (2.08-2.01)^a^
*R*_sym_ or *R*_merge_	0.034 (0.070)
*I*/σ*I*	24.2 (11.6)
Completeness (%)	95.1 (92.1)
Redundancy	2.6 (2.6)
**Refinement**	
Resolution (Å)	24.59-2.01
No. of reflections	35,510
*R*_work_/*R*_free_	19.1/23.8
No. of atoms	
Protein (A/B/D/G)	926/936/928/925
V5 (E/C/F/H)	c
Water	288
*B*-factors (Å^2^)	
Protein (E/B/C/D)	30.9/30.8/30.5/30.6
V5	28.0/28.7/29.1/29.0
Water	30.1
R.m.s.ds	
Bond lengths (Å)	0.009
Bond angles (°)	1.136

a Values in parentheses are for the highest-resolution shell.

### Maturation of the NbA1

A primary objective driving our research was to advance the study of PPIs utilizing a versatile nanobody-based sensor. While NbA1 demonstrates intracellular functionality, it exhibits a marked preference for the C-terminally tagged β-arrestin-2. This partiality can potentially be attributed to the lack of interactions with the last two residues of the V5 tag. To improve the overall affinity, particularly for the N terminus of the V5 tag, *in silico* affinity maturation was performed using the Rosetta modeling software. The generated models were assessed based on their corresponding scores and binding energy metrics, resulting in 10 designed mutants harboring degenerated mutations, which were subsequently synthesized and tested using the Tango assay. Based on the functional evaluation, it was deduced that two mutations (ΔD59 and S60K) resulted in better activity than NbA1, especially for the N-terminal V5-tagged arrestin. Consequently, an optimized nanobody was generated to incorporate only those two mutations, called NbV5. As shown in Figure 3*E*, the maturated NbV5 can recognize both N- or C-terminally tagged β-arrestin-2. In the case of AT1R, no discernible disparity was observed between the two orientations of the tag. However, in the context of μ-OR, a slight reduction in the recognition of β-arrestin-2-V5 was noted; nevertheless, this was compensated by a significant gain of function towards V5-β-arrestin-2 (N-terminal tag) (Fig. 3*E*). Although the precise mode of interaction remains to be elucidated, this optimized NbV5 variant exhibited a notable improvement in its overall behavior within our functional Tango assay, warranting its selection for further development of our nanobody-based biosensors.

Determination of the apparent KD of NbV5 was carried out using yeast surface display in the EBY100 yeast strain. Using the Gap-repair method, NbV5 was cloned in a yeast display vector (pYD1-Halo) derived from pCTCON2 (64), resulting in the construct NbV5-AGAP2-Halo-myc in N-terminal to C-terminal orientation. Efficient display of the nanobody was verified using flow cytometry (FACS), utilizing Halo-Alexa 488 as the labeling agent (Fig. S2, *A*–*C*). As for the antigen, we opted for the 1N3R Tau protein isoform (NP_001190180.1) with a V5 insertion between E73 and A74 and labeled with Alexa Fluor 488 succinimidyl ester. This unstructured protein is well-suited for displaying linear epitopes (65), avoiding any bias related to the N-terminal or C-terminal Tag-V5 presentation. Assessment of binding was conducted by FACS, specifically using serial dilutions of labeled Tau-V5 ranging from 500 nM to 0.98 nM. The findings, as presented in Fig. S2*D*, indicated an estimated affinity of 29 nM for NbV5. Nevertheless, it is important to emphasize that *in vitro* affinity measurements may not accurately reflect cellular affinity or activity.

### Protein–protein interaction assays

PPIs lie at the core of cellular processes, wherein there is a reciprocal and dynamic interplay between two proteins, often generalized as the "bait" and "prey". In our NbV5:V5 system, the bait represents a membrane receptor from the GPCR family. Notably, due to the inherent membrane configuration of GPCRs, their fusion is limited to the C-terminal region. Conversely, the prey protein represents any protein of interest tagged with the V5 epitope, which can be positioned at the C terminus, N terminus, or even within the protein sequence itself. As a proof-of-concept, we utilized the well-established GPCR interactors, β-arrestin-1 and β-arrestin-2, as the prey proteins. Within this tripartite system, the NbV5-based biosensor acts as a molecular bridge, reconstituting a functional cell-based assay by orchestrating the interaction between the V5-tagged prey protein and the bait receptor, which carries the complementary functional moiety. Importantly, this setup ensures minimal disruption of the native complex formation and preserves the intrinsic functionality of the prey protein.

The initial characterization of our platform with GPCRs enabled tunable regulation of the interaction dynamics through the utilization of an agonist and, thus, a more precise assessment of biosensor efficiency. Subsequent experiments were conducted using two distinct GPCRs, namely the AT1R and μ-OR, both of which are widely studied receptors in our laboratory and thus would serve well as targets for the characterization of the Nb-V5 biosensors.

### NbV5-based protease-dependent reporter assay (Tango)

The first functional assay adapted as a tripartite NbV5:V5 system was the Tango assay. Renowned for its high sensitivity and permissibility, previous efforts have successfully exploited this platform to investigate β-arrestin recruitment at specific GPCRs or to conduct high-throughput screenings of the entire class A GPCR-ome simultaneously (66, 67, 68). The Tango assay leverages a modified GPCR, termed Tango-ized GPCR, which carries a transcription factor, tetracycline-controlled transactivator (tTA), fused at its C terminus and preceded by a TEV endopeptidase cleavage site. Upon recruitment of the β-arrestin protein fused with TEV protease (β-arrestin-TEV) to the receptor, the TEV protease cleaves its recognition site, liberating the tTA transcription factor. Subsequently, the translocated tTA engages the nucleus and activates a reporter gene under the control of the tTA-response element. Our study selected an optimized version of the TEV protease, TEV219, which exhibits enhanced efficiency and avoids self-inactivation (69), to improve further the GPCR-TANGO platform’s performance (70).

**Figure 4:**
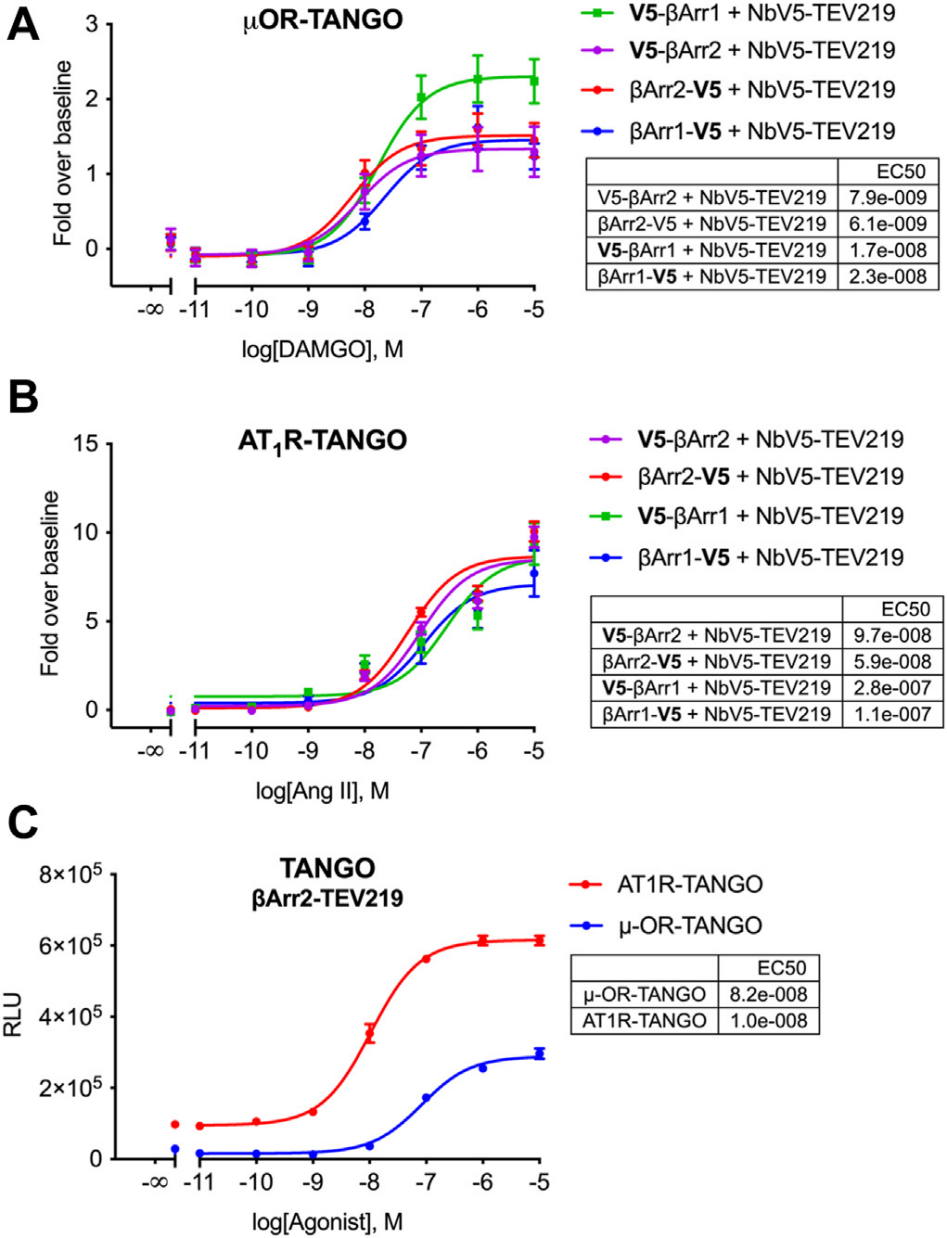
**NbV5 as a versatile nanobody-based biosensor: application in protease-dependent cell-based assay (TANGO).** The μ-OR-TANGO (*A*) and AT_1_R-TANGO (*B*) were cotransfected with β-arrestin1 or β-arrestin2 carrying a C- or N-terminal V5-peptide tag along with the NbV5-TEV219 fusion protein. Dose-response agonist treatments demonstrate the recruitment of β-arrestin in all configurations tested. *C*, the μ-OR-TANGO and AT_1_R-TANGO were cotransfected with β-arrestin2-TEV219 and stimulated with agonists showing that EC50 obtained using the original TANGO is similar to the NbV5-adapted TANGO. Dose-response curves were built using XY analysis for nonlinear regression curve and the 3-parameters dose-response stimulation function from GraphPad Prism. Baseline corrected curves were constructed using the “Remove baseline and column math” function (Value-Baseline/Baseline). Wells in the absence of ligand were used as the baseline for each condition. All error bars represent SD of three or four technical replicates. Data presented are representative of three biological replicates. μ-OR, mu-opioid receptor; AT1R, angiotensin II receptor type 1; TEV, tobacco etch virus.

Assessment of the NbV5 biosensors within the Tango assay was conducted using μ-OR and AT1R. Interestingly, a robust dose-response relationship was observed for both β-arrestin-1 and β-arrestin-2, regardless of the position of the V5-tag (Fig. 4, *A* and *B*). It is noteworthy to highlight that the obtained EC50 values were comparable to those obtained from our updated Tango assay featuring a direct fusion of TEV219 at the C terminus of the β-arrestins (Fig. 4*C*). The interassay reproducibility of the Tango assay is presented in Fig. S3*A*, where data from multiple biological replicates were normalized toward the top-performing conditions and pooled. These findings substantiate the suitability and efficacy of our NbV5 biosensors in conjunction with the TANGO-based assay, further affirming their potential as valuable tools in PPI studies.

### NbV5-based BRET

**Figure 5:**
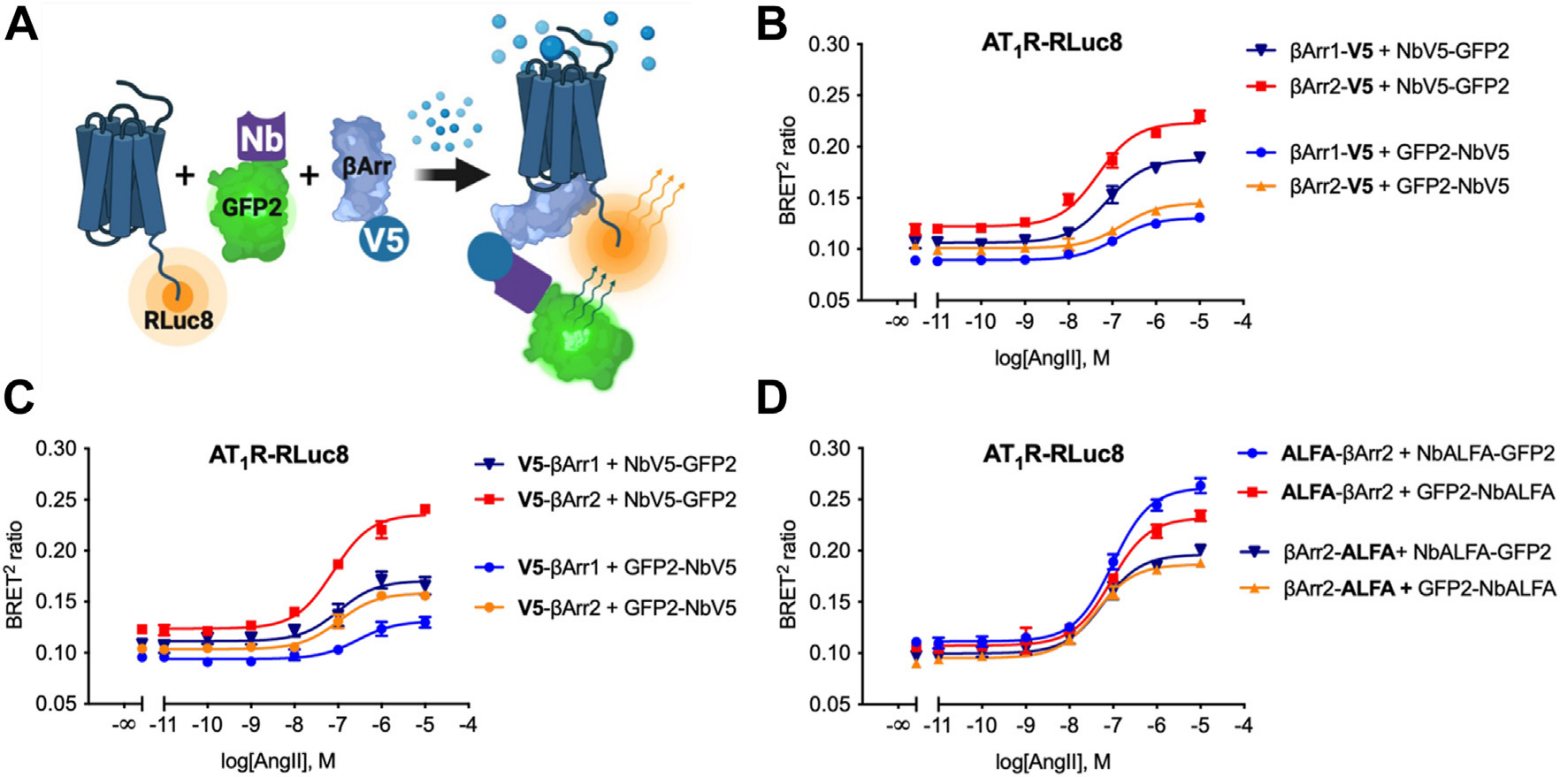
**NbV5 as a versatile nanobody-based biosensor: application in *B*ioluminescence *R*esonance *E*nergy *T*ransfer (BRET**^**2**^**).***A*, schematic of the BRET^2^ cell-based assay used to evaluate the NbV5. The recruitment of the β-arrestin–NbV5-GFP2 complex to the receptor fused to RLuc8 allows the energy transfer between the *Renilla reniformis* Luciferase mutant (RLuc8) and the GFP mutant GFP2 in the presence of the RLuc8 substrate Coelenterazine 400a. *B* and *C*, NbV5-based detection of β-arrestin1 and β-arrestin2 recruitment at the AT_1_R-RLuc8. N- and C-terminally V5-tagged β-arrestins were tested as well as both N- and C-terminally GFP2-tagged NbV5. Dose-response curve treatment with angiotensin II reveals equivalent recruitment of β-arrestin1 and 2 at the AT_1_R. *D*, similar results were obtained with the nanobody that recognizes the synthetic ALFA-tag (NbALFA). Dose-response curves were built using XY analysis for nonlinear regression curve and the 3-parameters dose-response stimulation function. All error bars represent SD of three biological replicates with two technical replicates (n = 6). AT1R, angiotensin II receptor type 1.

Biophysical techniques involving resonance energy transfer enable monitoring of the formation of dynamic complexes in living cells (71), with their use being well-established and remaining a gold standard in GPCR signaling (8). In the second generation of BRET (BRET2), the receptor is fused at the C terminus with the Renilla Luciferase variant 2 or 8 (RLuc2 or 8) (72), while the nanobody is fused at either N terminus or C terminus with the GFP2 acceptor (Fig. 5*A*) (73, 74). The underlying principle involves the generation of a resonance energy transfer signal when there is sufficient spectral overlap between the acceptor and donor molecules in close proximity, leading to dipole-dipole coupling. Importantly, the extended working distance range afforded by the BRET2 system enables the study of larger protein systems, including GPCR-protein interactor assemblies.

In a manner analogous to the adaptation of the TANGO system, various configurations involving β-arrestin-1 and β-arrestin-2 fused at the N terminus or C terminus with the V5-tag were assessed, alongside the reference ALFA-tag, which served as a benchmark due to the well-characterized performance of the corresponding NbALFA intrabody with different tag placements (N-term, C-term, and internal). As depicted in Figure 5, *B*–*D*, all configurations exhibited a robust signal; however, it was observed that the C-terminally tagged NbV5 (NbV5-GFP2) yielded a superior BRET2 ratio, while the impact on NbALFA was comparatively less pronounced. One possibility behind this intriguing observation could be explained by the distinct binding orientation of NbALFA, which is oriented at a 90-degree angle relative to NbV5, as illustrated in Fig. S1. Notably, the tag placement within the prey protein did not substantially influence the receptor-β-arrestin coupling under investigation. For the AT1R receptor specifically, both isoforms of β-arrestin were well recruited, with a slight preference observed for β-arrestin-2 within this particular assay. However, it is worth highlighting that we failed to detect a significant β-arrestin BRET signal at the μ-OR receptor, even in the presence of GRK2. While BRET has frequently been employed to quantify β-arrestin2 recruitment at the μ-OR receptor, it is noteworthy that the BRET ratio is always weaker than other GPCRs, including the closely related kappa opioid receptor, which worked in our Nb-V5–adapted BRET assay. The reasons underlying the ineffectiveness of the μ-OR receptor in this assay remain unclear; however, we speculate that the overall conformation of the μ-OR receptor may not be conducive to efficient resonance energy transfer. Notwithstanding this observation, we successfully detected β-arrestin recruitment utilizing the other two assays presented in this study. Consequently, our NbV5-based biosensors demonstrate substantial promise for implementation in BRET assays, but adding a tripartite constituent might not be favorable to efficient resonance energy transfer with specific pairs.

### NbV5-based NanoBiT

The NanoBiT assay represents a versatile structural complementation reporter system, harnessing the unique properties of a LgBiT (18 kDa) subunit and its corresponding Small BiT (SmBiT; 11 amino acids) peptide, specifically engineered to exhibit a low affinity for LgBiT. When these two proteins interact, the subunits assemble into an active enzyme which, in the presence of its substrate, furimazine, produces a luminescent signal in living cells, enabling real-time kinetic measurements of protein interaction dynamics (75). In the context of GPCRs, it is conventionally advantageous to fuse the SmBiT peptide to the C terminus of the receptor, likely minimizing perturbations to the receptor’s native state or ligand-induced complexes.

**Figure 6:**
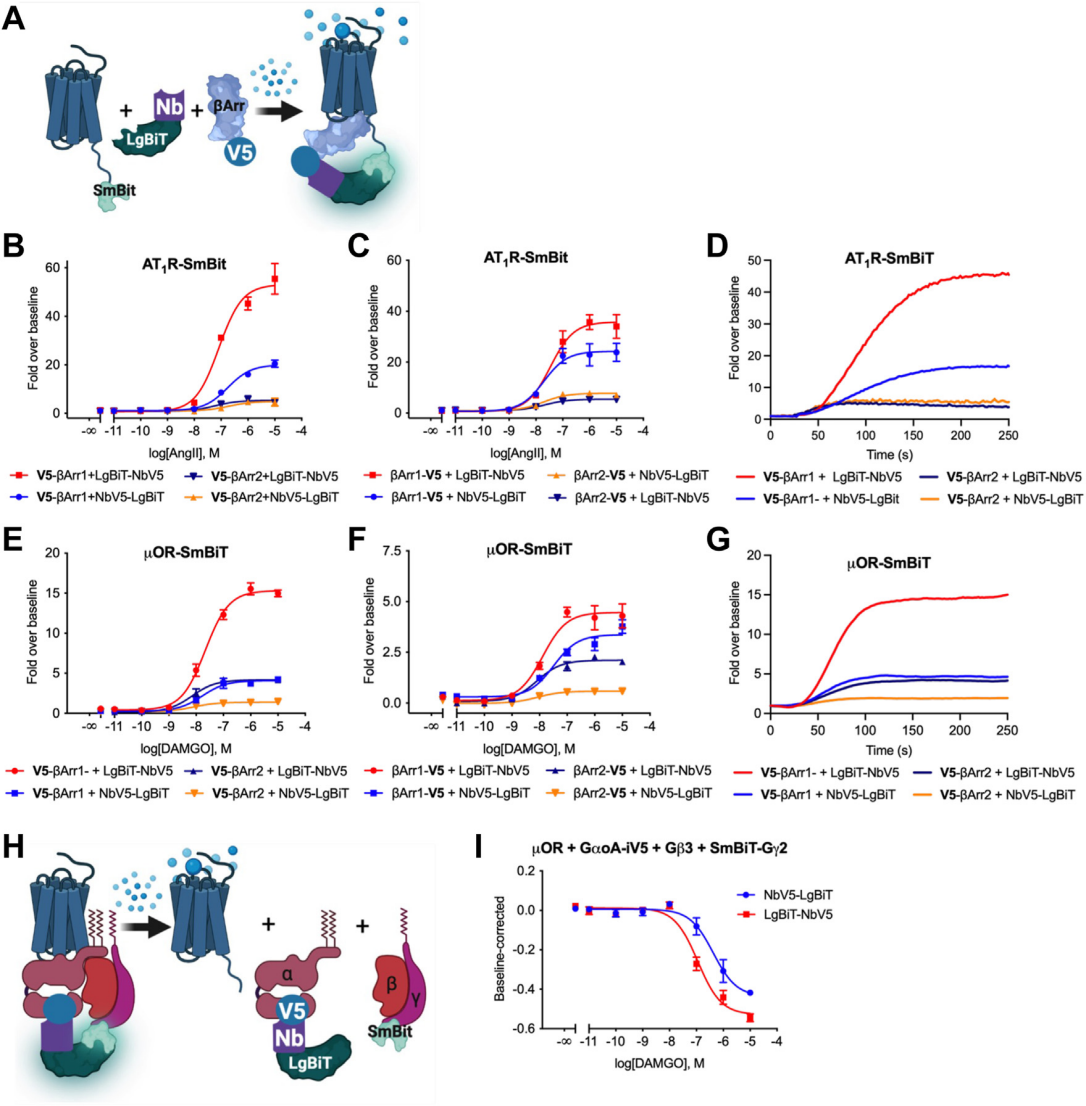
**NbV5 as a versatile nanobody-based biosensor: application in *Nano*luciferase *Bi*nary *T*echnology.***A*, schematic of the NanoBiT cell-based assay used to evaluate the NbV5. NbV5-based detection of β-arrestin1 and β-arrestin2 recruitment at the AT_1_R-SmBiT (*B*–*D*) and μ-OR-SmBiT (*E*–*G*) was assayed. N- and C-terminally V5-tagged β-arrestin was tested as well as both N- and C-terminally LgBiT-tagged NbV5. *D* and *G*, live kinetic trace at 1 μM agonist are shown in (*D*) for the AT1R and (*G*) for the μ-OR. The trace represents the mean of a quadruplicate experiment. *H*, schematic of the NanoBiT cell-based assay used to assess the internal V5-tag. *I*, functional recognition of an internal localized V5-tag was also assayed by NanoBiT by incorporating the V5-tag at position 92 of the G⍺oA (G⍺oA-iV5) and measuring the dissociation from the Gβ3 and SmBiT-Gɣ2 dimer upon μ-OR activation with DAMGO. *B*, *C*, *E*, *F*, and *I*, dose-response curves were built using XY analysis for nonlinear regression curve and the 3-parameters dose-response stimulation function from GraphPad Prism. Baseline correction was performed using the “Remove baseline and column math” function, calculated as Value-Baseline/Baseline (*B*–*G*) or Value-Baseline (*I*). Wells in the absence of ligand were used as the baseline for each condition. All error bars represent SD of three or four technical replicates. Data presented are representative of three biological replicates. μ-OR, mu-opioid receptor; AT1R, angiotensin II receptor type 1; LgBiT, Large BiT; NanoBiT, Nanoluciferase Binary Technology; SmBiT, Small BiT.

As depicted in Figure 6, various configurations of β-arrestin fusions (V5-β-arrestin and β-arrestin-V5) and nanobody fusions (Nbs-LgBiT and LgBiT-Nbs) were compared to evaluate their respective functional performances. In contrast to the BRET2 experiment (Fig. 5), LgBiT-NbV5 produced a superior response than NbV5-LgBiT, underscoring the importance of evaluating both orientations to achieve optimal response and sensitivity. Interestingly, the NanoBiT assay revealed a pronounced preference for β-arrestin-1 over β-arrestin-2 at the AT1 receptor, a trend also observed with the ALFA-tag system, thereby confirming that the choice of tag system does not significantly influence the outcomes (Fig. S2*A*). To detect reasonable recruitment at the μ-OR receptor, the co-expression of GPCR kinase 2 (GRK2) is often necessary, particularly considering its absence at endogenous levels in human embryonic kidney 293 (HEK293) cells (76). As shown in Figure 6, *E* and *F*, β-arrestin-1 was favored over β-arrestin-2 at the μ-OR receptor, with the N-terminally V5-tagged β-arrestins and N-terminal LgBiT-NbV5 identified as the optimal orientations for tripartite interaction at this receptor. Fig. S3*B* displays the interassay reproducibility of the NanoBiT assay, wherein data from various biological replicates were normalized relative to the most optimal conditions and then pooled.

The NanoBiT assay offers notable advantages, particularly its suitability and ease for performing live experiments. While it can be employed for endpoint assays on a microplate reader, using an appropriate real-time kinetic reader, such as the Fluorescent Imaging Plate Reader-TETRA, can reveal additional information in live settings. This aspect holds particular relevance for GPCRs, as it is often assumed that all interactions occur under equilibrium conditions, despite it being well-established that the interactions are transitory in nature, especially in the case of heterotrimeric G proteins (77). Thus, to study GPCR signaling under nonequilibrium conditions, kinetic experiments and appropriate models should be favored (78). In light of this, we present a live kinetics experiment in Figure 6, *D* and *G*, wherein the recruitment of V5-tagged β-arrestin at the μ-OR and AT1R receptors is examined at a single concentration of 1 μM selective agonist.

The ability of NbV5 to recognize an internally positioned V5-tag was also evaluated, specifically using the inhibitory G protein G⍺oA with the V5-tag inserted at position 92. It is well established that inhibitory G proteins tolerate such insertions, commonly employed for the introduction of fluorescent proteins or RLuc8 for BRET experiments. Figure 6*H* illustrates the employed approach, where SmBiT was fused at the N terminus of the G*γ*2 subunit and co-expressed with Gβ3, thereby facilitating the assembly of the obligatory Gβγ dimer that interacts with the inactive GDP-bound G⍺oA. Upon activation by the G⍺i/o-coupled μ-OR receptor, G protein activation can be detected through the dissociation of the heterotrimer. As depicted in Figure 6*I*, activation of the μ-OR receptor by its agonist, DAMGO, induces the dissociation of the trimer, which can be monitored using NbV5 fused with LgBiT at the N terminus or C terminus. Hence, our Nb-based sensor enables the measurement of G protein activation through the internal insertion of the V5 sequence, thereby minimizing the potential impact on overall G protein regulation. Furthermore, based on previous experiments conducted in our laboratory, it has been determined that NanoBiT complementary subunits are not well-tolerated when placed internally, further reinforcing the advantage of utilizing the NbV5:V5 tag system.

With regard to epitope tags, these incorporations are highly favorable as they avoid the need to generate custom antibodies for each protein of interest and allow orthogonal peptide tags to be multiplexed within the same experiment (11, 13); thus, the selectivity of the nanobody toward its target peptide tag is of paramount importance. As demonstrated in Fig. S4*B*, NbV5 exhibits remarkable specificity for the V5 epitope while displaying no detectable activity towards the ALFA-tag. This finding underscores the potential for simultaneous utilization of both tags broadening the experimental possibilities of our nanobody-based framework. To illustrate the application for a multiplexing experiment, the C-terminally V5-tagged AT1R was used to bridge the NbV5 carrying the SmBiT to the LgBiT-tagged NbALFA, detecting the recruitment of ALFA-tagged β-arrestin1. All combinations were permissive, but the SmBiT-NbV5 resulted in the best fold-over baseline compared to NbV5-SmBiT. Interestingly, while the fold-over baseline was substantially reduced, a gain into EC50 was observed from ∼10 nM to ∼1 nM (Fig. S4*A* vs. Fig. S4*C*). We do not have a definitive explanation for the observed increase, as the SmBiT-tag (11 aa) is approximatively the same size as the V5-tag (14 aa). However, the higher affinity of both Nbs for their respective tags compared to SmBiT–LgBit interaction could account for this gain or alternatively, it could be attributed to a more favorable spatial orientation of the complex. Together, Fig. S2, *B* and *C* strongly support the selectivity of each tag system and their application for multiplexing configurations.

### Nbs as fluorescence trackers for microscopy

**Figure 7:**
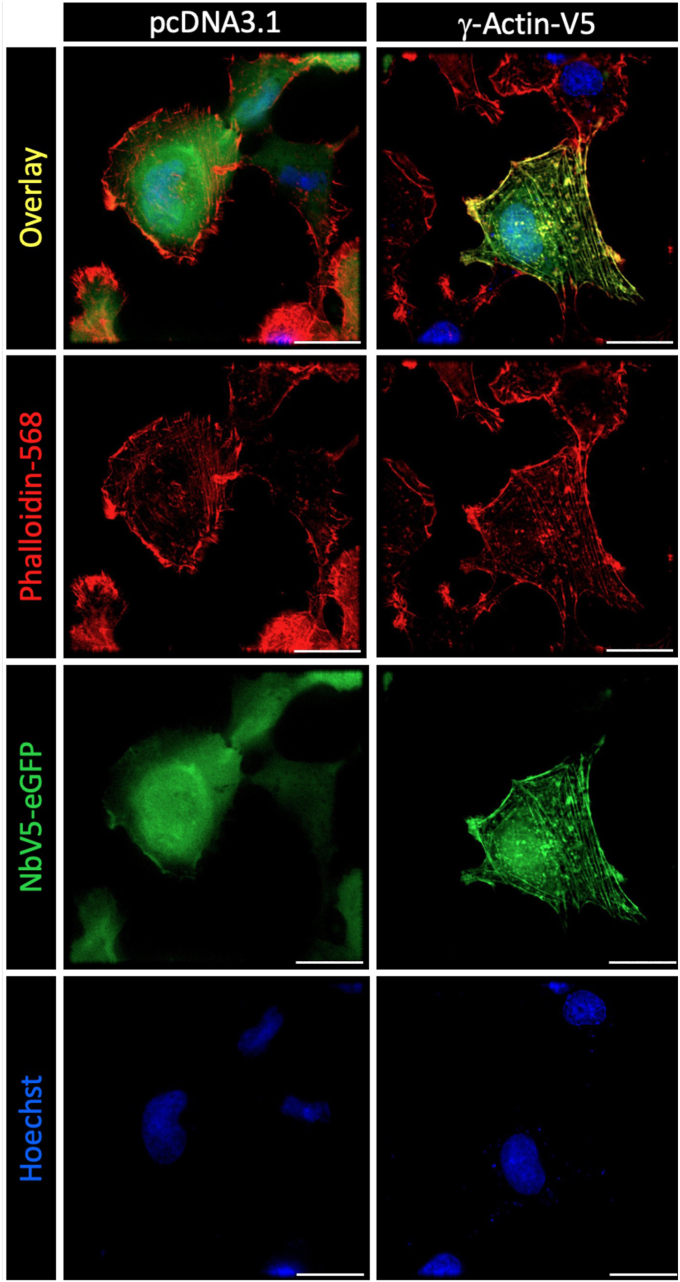
**NbV5-based detection of V5-tagged proteins by cell imaging.** Fluorescence imaging of HT1080 cells expressing NbV5-eGFP and ɣ-Actin-V5 or pcDNA3.1+ as a negative control. In the absence of ɣ-Actin-V5, NbV5-eGFP (*green*) is diffused throughout the cell, while in the presence of ɣ-Actin-V5, NbV5-eGFP is enriched in actin-rich protrusions and structures. Cells were costained with the F-actin probe Alexa Fluor 568 phalloidin (*red*) and the blue DNA stain Hoechst. The overlay is shown on the *top panel*. Images are representative of 25 cells from three independent experiments. Scale bars represent 25 μm.

To explore the potential application of NbV5 as a genetically encoded tracker, we conducted experiments involving the co-expression of *γ*-actin tagged with a V5 epitope at its C terminus (*γ*-actin-V5) along with NbV5 fused with the constitutive fluorescent protein eGFP. As shown in Figure 7, the NbV5-eGFP fusion protein diffuses within the cell when expressed with the control pcDNA3.1 but selectively associates with *γ*-actin-V5 in its presence. Importantly, discernable actin-related structures like stress fibers and lamellipodia can be observed, indicating that the NbV5-eGFP (green) could efficiently interact with a dynamic protein such as *γ*-actin without interfering with its natural localization. The selectivity of the interaction is shown by the colocalization (yellow) between *γ*-actin-V5:NbV5-eGFP staining (green) and direct staining using the phalloidin-568 (red), which is a high-affinity F-actin probe. While the phalloidin selectively stains the actin filaments (F-actin), the *γ*-actin-V5:NbV5-eGFP also stains the globular actin (G-actin). Therefore, NbV5-based biosensors could be used to track protein remodeling or trafficking in a live cell-based experiment.

## Discussion

Existing biotechnological methodologies used for the characterization and validation of putative drug targets and as the elucidation of physiological and pathophysiological cellular processes necessitate a wealth of information pertaining to the abundance, localization, and dynamic interactions of cellular constituents (79). Consequently, the development of intracellular traceable proteins for both endpoint investigations and real-time kinetics diagnostics assumes paramount significance, particularly in light of increasing efforts to acquire a more comprehensive understanding of cellular PPIs and thus extending the applicability of such intracellular tracers to encompass a genome-wide even to an organism (-omics-) scale.

In this study, we contribute to this endeavor by developing and characterizing a V5-tag–targeted nanobody named NbV5 alongside a suite of NbV5-based intracellular biosensors with multipurpose functionalities. The premise of our approach was to avoid the fusion of a large, functionalized tag to a protein while taking advantage the vast repertoire of C-terminally V5-tagged genome-wide open source ORFs. The V5 epitope, an established and widely employed peptide tag for over 3 decades, assumes a pivotal role in our investigation. This 14-mer peptide is devoid of net charge under physiological pH conditions and does not have any predicted secondary structures, albeit featuring quite an ordered core owing to the presence of three proline residues. The inherent rigidity is posited to reduce the multidimensional complexity of the conformational landscape, thereby mitigating the impact of environmentally induced secondary structures and conferring stability in solution (80).

Multiple peptide tags have emerged, each bearing its own set of advantages and disadvantages, yet few tags have corresponding functional intrabodies that are thoroughly characterized, as done in this study. While it is worth noting the exceptions of the NbALFA:ALFA-tag (51) and Moon-tag (46, 47), it is crucial to highlight that neither of these were thoroughly characterized for PPI studies as showcased herein with the NbV5; nonetheless, the availability of these tag systems could capitalize on through simultaneous multiplexing implementations in conjunction with the NbV5:V5. Unique to the V5-tag is the associated commercially available V5-tagged genome-wide ORF library, thereby paving the way for the prospect of large-scale PPI investigations. Furthermore, it must be underscored that among the numerous available nanobody structures, only a handful involve those complexed with linear peptide tags, namely ALFA (PDB: 6I2G), BC2 (PDB: 5IVO), and the V5 tag (reported herein). One avenue for future investigation is evaluating whether advanced protein modeling programs, such as Alphafold2, Alphafold Multimer, or IgFold, can provide accurate predictions of nanobody-peptide tag structures (81), which would help inform the development of novel optimize future nanobody scaffolds with an increased propensity for intracellular stability. Recently, Dingus *et al*. have developed a general consensus framework derived from the most highly conserved positional residues across a large group of intracellularly stable Nbs, which would also be a valuable asset for increasing the intracellular stability of Nbs through targeted mutagenesis without compromising target binding (15).

The majority of Nbs available to date have been developed by animal immunization, but the utilization of synthetic *in vitro* platforms, as exemplified in our study, holds immense promise. The Hybribody methodology employed combines peptide phage display and intracellular Y2H techniques, as well as the selection of Nbs wherein the folding of the paratope is independent of disulfide bonds, thus significantly enhancing the enrichment and identification of viable intrabodies (60, 82). This screening approach isolated a cohort of prospective Nbs, among which clone NbA1 emerged as the preeminent candidate as an intrabody. Initial investigations unveiled a low expression level for all Nbs, circumvented through codon optimization tailored for a human expression system. Subsequent characterization in the Tango assay revealed that NbA1 exhibited suboptimal recognition of the N-terminally positioned V5-tag on β-arrestin2. To address this limitation, we sought to resolve its crystal structure, which would provide insight into the binding interface of NbA1:V5 tag. Consequently, NbA1 was purified from the periplasm of SHuffle bacteria, which are best suited for the purification of proteins containing disulfide bonds (83), and the purified NbA1 was then cocrystallized with the V5-tag. The resultant crystal structure served as the foundation for computer-aided maturation, aimed at refining the functionality of the nanobody. The maturation process was confined to the three CDRs, with no discernible mutants predicted to induce significant changes in binding energy. Based on the mutants tested in Tango experiments, it was determined that two specific mutations conferred improved recognition of the V5-tag. The key distinguishing feature of NbV5 vis-à-vis NbA1 lies in its interaction with the N-terminally situated V5-tag, as shown in Figure 3*E*. Given that these two key mutations, ΔD59 and S60K, are juxtaposed within a disordered loop in CDR2 (Fig. S1), the deletion of a single residue is postulated to confer stability to this region, while Lys60 establishes direct contacts with the CDR2 loop, thereby further stabilizing this region (Fig. S1, *B* and *C*). While it is generally accepted that CDR3 is the most critical region for antigen recognition and binding due to its longer length and higher variability, several reports and as demonstrated herein demonstrate that mutations in CDR1 or CDR2 may optimize affinity or stability, particularly if these regions are directly involved in the paratope interaction (84). Thus, comprehensive optimization strategies should involve exploring mutations in all three CDRs to assess their combined impact on nanobody performance.

Having delineated the general mode of interaction between the nanobody and V5-tag, we proceeded to construct NbV5-based biosensors for versatile applications in the most widely used cellular PPI assays, namely Tango, BRET, and NanoBit assays. As shown herein, our biosensors probed the recruitment of both β-arrestin-1 and β-arrestin-2 at two well-characterized GPCRs, the μ-OR, and the AT1R. The μ-OR serves as the primary target for the most prescribed analgesics such as morphine and fentanyl (85). As for AT1R, this receptor is important for controlling vasocontraction, with AT1R antagonists (ARBs) being prescribed for the treatment of hypertension, congestive heart failure, and diabetic nephropathy (86). Although both receptors are known to engage both β-arrestin isoforms, there exists conflicting data regarding isoform selectivity and, more importantly, the intrinsic drug efficacy. As the measurement of the direct interaction between the receptor and its effector is a key aspect for accurately determining intrinsic drug efficacy (87), the use of smaller tags should mitigate some distortions caused by larger functional tags. With regard to GPCRs, the fusion of a large moiety to its C terminus may introduce artifacts that lead to impaired signaling, altered ligand binding, mislocalization and affect trafficking, and changes to their stability and expression levels, likely due to steric hindrance or altered receptor conformation caused by the GFP fusion (88). Therefore, tag systems such as V5 would enable a more faithful characterization of receptor–effector interactions.

This work’s adaptation of common cellular-based PPI assays involved the integration of NbV5-based biosensors, commencing with the TEV-dependent reporter assay (Tango), given its permissibility and sensitivity. Subsequently, our efforts extended to the adaptation of two widely employed PPI assays, namely BRET and NanoBiT. Intriguingly, a discrepancy between BRET and NanoBiT assays surfaced when investigating the selective recruitment of β-arrestin isoforms at the AT1R receptor, with a distinct preference for β-arrestin-1 over β-arrestin-2 observed solely in the NanoBiT assay, while BRET2 and TANGO assays yielded no such disparity. The underlying reasons behind this observation remain unknown. Still, it is plausible to consider that the size and orientation of the tag may exert differential influences contingent upon the assay employed. In essence, these effects could manifest as alterations in the kinetics of PPIs or literal steric hindrance during complex formation. Another layer of complexity emerges from the intrinsic principles governing each assay: BRET, being highly conformationally dependent, necessitates optimal distance and spatial orientation for effective dipole-dipole coupling to facilitate efficient resonance energy transfer (89). One plausible explanation is that the measured BRET2 signal represents an average of recruitment and conformational changes (multistates). Conversely, in binary complementation assays like NanoBiT, the two complementary fragments must assume a favorable spatial arrangement to facilitate complex formation. Moreover, binary complementation assays solely capture newly recruited β-arrestin or a single conformational state of the complex, precluding the monitoring of spatial rearrangements (90). In the case of Tango, the prolonged stimulation and slower kinetics, due to the inherent proteolytic activity of TEV (69), might mitigate any detrimental kinetic effects stemming from tag size or orientation, albeit steric hindrance remains a pertinent consideration. While the implementation of a tag system has the potential to alleviate the impact of steric hindrance or particular arrangements of interacting partners during complex formation, the tag system itself may influence the sensitivity of the assay under specific circumstances. This phenomenon was evidenced within our system, as NbV5 failed to detect arrestin recruitment to the μ-OR in the BRET2 assay while performing well for AT1R. It should also be noted that during the optimization process, diverse sizes of flexible linkers (ranging from 5 to 70 amino acids) were explored to ensure the optimal distance between functional moieties (91); however, varying linker size did not improve the lack of detected at μ-OR. While a modest effect on the observed activity was noted at AT1R, the small (GGGGS)2× fusion linker was ultimately chosen as the optimal peptide for the NbV5-based biosensors.

While the current work primarily focused on utilizing GPCRs as a means to characterize the NbV5-based system, future efforts will be consecrated to expanding the applicability of the NbV5:V5 system to a broad range of PPIs, such as other membrane receptor–protein and protein–protein interactions. Thus, the authors hope that the versatility and adaptability of the NbV5:V5 platform will contribute to probing and unraveling the intricate dynamics of various interaction networks.

## Conclusion

Herein, we engineered a novel nanobody that recognizes the V5-tag epitope, a widely employed peptide tag in many expression vectors, including the MISSION TRC3 human genome-wide ORF library. Said nanobody was selected and maturated to optimize its functionality in the intracellular environment, culminating in the development of NbV5. Our NbV5 intrabody offers a broad range of applications, including biosensors in cellular-based PPI assays, such as NanoBiT, Tango, and BRET^2^, and for microscopic imaging purposes. Moreover, the potential of NbV5 extends beyond its current scope and can be further expanded to adapt other existing PPI assays. In summary, NbV5 represents a unique tool that offers traceability of intracellular binding proteins with minimal disturbance to the native cellular milieu.

## Experimental procedures

### Cell culture

HEK293T and HT1080 cells were obtained from the American Type Culture Collection and maintained in Dulbecco’s modified Eagle’s medium (DMEM) supplemented with 5% fetal bovine serum (FBS) (Thermo Fisher Scientific), 5% bovine calf serum (Thermo Fisher Scientific), and 1× Pen-Strep (100 U/ml penicillin and 100 μg/ml streptomycin) (Thermo Fisher Scientific). HEK293T cells stably expressing μ-OR-SmBiT (μ-OR-SmBiT/HEK293T) were generated by transduction with lentivirus particles, which had been produced in HEK293T cells by transiently cotransfecting the lentiviral packaging plasmid psPAX2 (Addgene #12260), a lentiviral vector encoding μ-OR-SmBiT (pLenti-Blast Addgene #17451), and the envelope plasmid pCMV-VSV-G (gift from Marceline Côté) at a 1:1:1 ratio using PEI transfection reagent. The following day, media was changed, and the supernatant was harvested at 48h posttransfection. Cells were then transduced with lentivirus in standard growth media containing 5 μg/ml polybrene and the next day, selected with blasticidin at 5 μg/ml. All cells were cultured at 37 °C in a humidified atmosphere containing 5% CO_2_.

### Plasmids and cloning

All plasmid DNA used in this publication were fully sequenced and are available upon request or through Addgene repository (https://www.addgene.org/Patrick_Giguere/). Plasmid encoding ɣ-Actin-V5 was extracted from the MISSION TRC3 Human LentiORF Puromycin library (MilliporeSigma). V5-βArrestin1, V5-βArrestin2, βArrestin1-V5, βArrestin2-V5, βArrestin2-ALFA, ALFA-βArrestin2, βArrestin1-ALFA, and ALFA-βArrestin1 were amplified by PCR, including the V5 tag within the primer, and subsequently cloned into pcDNA3.1^+^ at the HindIII-XbaI restriction sites. GɑoA with internal V5-tag at position 92 was synthesized by IDT (Integrated DNA Technologies) and cloned at HindIII-XbaI sites in pcDNA3.1^+^. BRET^2^ constructs: AT_1_R-RLuc8, μ-OR-RLuc8, NbV5-GFP2, GFP2-NbV5, βArrestin2-GFP2, βArrestin2-ALFA-GFP2 were amplified by PCR and cloned in pcDNA3.1^+^ using NEB HiFi DNA Assembly (New England Biolabs). Gβ3 and Gɣ2-GFP2 were generously gifted by Dr Asuka Inoue (TOHOKU University). NanoBiT constructs: μ-OR-SmBiT and AT_1_R-SmBiT were amplified by PCR by including the SmBiT tag within the primer preceded by a (GGGGS)_2x_ linker. NbV5-LgBiT, LgBiT-NbV5, NbALFA-LgBiT, and LgBiT-NbALFA were amplified by PCR and cloned in pcDNA3.1^+^ using NEB HiFi DNA Assembly (New England Biolabs). Gɣ2-SmBiT was generously gifted by Dr Asuka Inoue (TOHOKU University). TANGO constructs: μ-OR-TANGO and AT_1_R-TANGO are from the original PRESTO-TANGO library (29, 30, 31). Aforementioned Nbs fused to the TEV219 were cloned by PCR at restriction sites HindIII-BamHI in pcDNA3.1^+^-X-TEV219 vector. NbV5-eGFP was generated by PCR amplification of NbV5 and cloned into pEGFP-N1 (Clontech) at the HindIII-BamHI sites.

### Nanobody development

To identify Nbs that bind to a linear target and that could be expressed from inside the cell (as intrabodies), phage display selection was conducted with the Nali-H1 synthetic library (57), by Hybrigenics Services (Paris, France), using a His-Halo protein fused with three successive V5-tags (Halo-3xV5). The naïve library was first depleted using another antigen fused in the same Halo vector and selected on magnetic streptavidin beads with the biotinylated Halo-3xV5. With the first round presenting a complexity of 3 × 10^6^ colonies, the DNA extracted from the first round were used to construct a Y2H prey library by PCR and Gap repair. The VHH selected after one round of phage display against V5 tag-biotin were cloned into the pP9 yeast prey vector, which is derived from the original pGADGH plasmid; the library had 2.6 x 10^5^ independent clones in yeast. A single V5 tag, as well as two tandem V5 tags, were cloned into pB27 as a C-terminal fusion to LexA (LexA-V5); pB27 is derived from the original pBTM116 plasmid (92). The constructs were verified by sequencing the insert and used as baits to screen the V5-specific VHH library. For the screen, clones were vetted using a mating approach with YHGX13 (Y187 ade2-101:loxP-kanMX-loxP, matα) and L40ΔGal4 (mata) yeast strains as previously described (59). Moreover, the library has been screened at saturation by cell-to-cell mating (59). A total of 264 His+ colonies were selected on a medium lacking tryptophan, leucine, and histidine supplemented with 0.5 mM 3-AT, obtaining 52 different VHH with redundancies from 1 to 37.

### Protein purification

The NbA1 was cloned into the expression vector pET26b (+) (Novagen) at NcoI-XhoI restriction sites to generate the pelB leader-NbA1-His_6_ construct. The plasmid was transformed in SHuffle T7 Competent *Escherichia coli* (New England BioLabs), and the NbA1 was subsequently purified from the periplasm of SHuffle T7 cells. Bacteria were then grown at 30 °C in Terrific Broth and, after reaching an A600 of ∼0.6 to 0.8, were induced with 1 mM IPTG (Thermo Fisher Scientific) at 25 °C for approximately 16 h. Bacteria were then pelleted and resuspended in a solution of lysis buffer (0.5 M sucrose, 0.2 M Tris pH 8, 0.5 mM EDTA) and water at a ratio of 1:2 to create an osmotic shock. The lysate was then frozen and thawed for more efficient purification. Following, the mixture was stirred for 45 min at 4 °C and brought to a concentration of 150 mM NaCl, 2 mM MgCl_2_, and 20 mM imidazole and centrifuged at 20,000*g* for 30 min at 4 °C. The supernatant was then filtered through a 0.22 μm filter. After filtration, the supernatant was added to a gravity column containing 4 ml of Ni-NTA (Qiagen). Beads were washed with a high salt buffer (20 mM Hepes pH 7.5, 500 mM NaCl, 20 mM imidazole) and washed three times with low salt buffer (20 mM Hepes pH 7.5, 100 mM NaCl, 20 mM imidazole). The Nbs were then eluted (20 mM Hepes pH 7.5, 100 mM NaCl, 400 mM imidazole) and dialyzed into physiological buffer solution (10 mM Hepes pH 7.4, 140 mM KCl, 10 mM NaCl) and purified using fast protein liquid chromatography (AKTA GE) on an S75 prep size-exclusion column.

### Crystallization, data collection, and structure determination

Purified NbA1 (30 mg/ml) was incubated with the V5 peptide in a 1:3 ratio (protein: peptide). The protein complex was crystallized *via* the sitting drop vapor diffusion method at 4 °C with a mother liquor composed of Bis-Tris pH 6.5, 19% (w/v) PEG 3350, and 20% (v/v) ethylene glycol. The crystals were flash-frozen in liquid nitrogen, and a full data set was collected using a Rigaku MicroMax-007HF equipped with a copper anode. Images were collected using an R-Axis IV++ detector (Rigaku) and processed using Structure Studio (Rigaku). The structure was solved by molecular replacement using the structure of NbALFA (PDB 6I2G) as search model and Phaser (50). Following several rounds of NbA1 building and refinement using COOT and Phenix, respectively, V5 was built in the positive Fourier map. The model was completed by adding the molecules and truncating side chains for which no electronic density could be observed. Ramachandran statistics: Nonglycine Ramachandran outliers; 0%, Nonglycine Ramachandran favored; 100%, Molprobity score: 1.65. Statistics of data collection and refinement are summarized in Table1. All structural figures were prepared in PyMOL.

### Nanobody maturation

To optimize the NbA1 sequence and potentially increase the affinity of the antibody towards V5, Rosetta single-state design protocol was performed using the Rosetta Software Suite on the crystal structure of the NbA1 complex. The structure of NbA1 was prepared for antibody affinity maturation for Rosetta by manually editing the PDB file in PyMOL. PyMOL command prompts were used to delete the unwanted water molecules and all nonessential ligands and chains. An extra processing step was also performed to remove any protein atoms that are not involved in the antibody-antigen interface; chains were also renamed and reordered to help differentiate the antibody residues from the antigen residues. Next, a resfile (python script) was generated to identify the residues that were within a distance of specified residues that define the NbA1 protein interface. The side chain conformations were optimized using the repacking and relaxing feature in Rosetta protein design, which was performed to minimize backbone phi-phi angles to relieve small clashes between side chains. The relaxed model was then used to generate 10 designed models through RosettaScripts XML file, which contains a design protocol that uses a single round of fixed backbone design. As a control, the same protocol was repeated to generate 10 control models without designing any residues. These control models were generated to compare the scores and the binding energies of the designed models to the native sequence during the analysis stage. The designed sequences were then analyzed by looking at the score, binding energy, and the binding density of the models. The analysis of the metrics was plotted using RosettaScripts which plots the score and the binding energy of the designed models against the control models. Subsequently, specific mutations were identified that resulted in the improvement of the NbA1 complex based on corresponding findings from functional assay experiments (as shown in Fig. 1). Finally, a sequence logo was generated from the designed models in order to determine which mutations were made and their frequencies.

### BRET^2^ assay

HEK293T cells were plated in 6-well plates at 1.2 × 10^6^ cells and subsequently transfected using the PEI precipitation method with BRET^2^ constructs at a total of 3 μg of DNA per well. Transfected cells were detached and seeded on poly-L-lysine (PLL)-coated white 96-well assay plates (Thermo Fisher Scientific). The following day, spent medium was removed and replaced with 60 μl of 1× Hanks’ balanced salt solution (HBSS) buffer, followed by the addition of 10 μl of Coelenterazine 400a (NanoLight Technologies) at 50 μM to each well, for a final concentration of 5 μM. After incubating the plates away from light for 8 min, 30 μl of serial dilutions of agonists at 3× concentration was added. Plates were subsequently read 4 times after 2 min, 10 min, 20 min, and 30 min using the Hidex Sense Beta Plus microplate reader (Gamble Technologies) with 405 nm (RLuc8-Coelenterazine 400a) and 500 nm (GFP2) emission filters, at 1 s/well integration times. Figures shown in the manuscript account for the reads after 20-min incubations with agonist (and correspondingly, approximately 30-min incubations with Coelenterazine 400a). Data were extracted using the integrated software and subjected to nonlinear least-squares regression analysis using the sigmoidal dose-response function provided in GraphPad Prism 9.0 (www.graphpad.com). Data of three independent experiments (N = 3) performed in quadruplicate are presented as BRET^2^ ratio (acceptor/donor) as indicated in figure legends.

### NanoBiT assay

μ-OR-SmBiT/HEK293T and HEK293T cells were plated in 6-well plates at 1.2 × 10^6^ cells and then transfected using the PEI precipitation method with the NanoBiT constructs the next day at a total of 3 μg of DNA per well. Transfected cells were detached and seeded on PLL-coated white 384-well assay plates (Thermo Fisher Scientific) in starvation media (DMEM, 1% FBS, 1× Pen-Strep), The next day, media was removed and replaced with 20 μl of 1× HBSS buffer containing 5 μM furimazine and incubated for a total of 10 min at room temperature before reading on Fluorescent Imaging Plate Reader Tetra system (Molecular Devices). Baseline measurements were initially read before drugs were added into their respective wells (concentration and different drugs in Figure legends). The subsequent changes in relative luminescence signals (relative luminescence unit, RLU) were recorded over time. Data were extracted using the integrated ScreenWorks software (www.moleculardevices.com/products/flipr-penta-high-throughput-cellular-screening-system/screenworks-software) and subjected to nonlinear least-squares regression analysis using the sigmoidal dose-response function provided in GraphPad Prism 9.0. Data of three independent experiments (N = 3) performed in quadruplicate are presented as RLUs or normalized as indicated in figure legends.

### Tango assay

HTTL (HEK293T stably expressing a luciferase reporter gene under the tTA-response element-Tight promoter) cells, an in-house developed reporter cell line (70), were seeded in 6-well plates at 1.2 x 10^6^ cells and were transfected with Tango-ized constructs using the PEI precipitation method. Twenty hours later, the transfected cells were plated in DMEM supplemented with 1% dialyzed FBS into PLL-coated 384-well white clear bottom cell culture plates at a density of 30,000 cells/well in a total volume of 40 μl for 5 h to ensure proper attachment of cells. Agonist solutions, previously prepared at 3× concentration in sterilized assay buffer (20 mM Hepes, 1× HBSS, pH 7.4), were added to the cells at 20 μl per well. Following overnight incubation, media was removed and 20 μl per well of homemade luciferase detection reagent (108 mM Tris–HCl; 42 mM Tris-Base, 75 mM NaCl, 3 mM MgCl_2_, 5 mM DTT, 0.2 mM coenzyme A, 0.14 mg/ml D-luciferin, 1.1 mM ATP, 0.25% v/v Triton X-100, 2 mM sodium hydrosulfite) was added to all wells (68). After 10 min of incubation in the dark at room temperature, plates were read using the Hidex Sense Beta Plus microplate reader (Gamble Technologies). Data were subjected to nonlinear least-squares regression analysis using the sigmoidal dose-response function provided in GraphPad Prism 9.0. Data of three independent experiments (N = 3) performed in quadruplicate are presented as RLUs or normalized as indicated in figure legends.

### Fluorescence imaging

HT1080 were seeded in an ibiTreat-chambered coverslip (Ibidi) in complete medium to obtain a 50% confluency the following day. The next day, cells were transiently cotransfected using JetPRIME (Polyplus Transfection) with ɣ-actin-V5/pLX307 or pcDNA3 and NbV5-eGFP/pcDNA3.1+. Twenty-four hours posttransfection, cells were fixed for 10 min in PBS containing 4% (w/v) paraformaldehyde, then washed three times in PBS, and incubated 30 min in PBS containing 0.1% Triton (v/v). The cells were then incubated with Alexa Fluor 568 Phalloidin (Thermo Fisher Scientific) (1:200 dilution) for 45 min and washed three times, followed by an incubation with 2 mM Hoechst 33342 (Thermo Fisher Scientific) for 15 min at room temperature. The coverslips were then washed with PBS, drained, and mounted with permafluor mounting media (Epredia). Cells were imaged on a GE Delta Vision Elite microscope using a 60×, 1.4NA, oil, Plan-Apo N objective. The images were analyzed using ImageJ software (https://imagej.net/ij/index.html).

### Affinity measurement

Yeast Display: pYD1-Halo is derived from the pCTCON2 plasmid while retaining the AGAP2 and Gal4 inducible system. The nanobody is first encoded into the strain EBY100 (64), followed by the Agap2 and the Halo-Tag (pHTC, Promega Corp). Preculture was performed in drop-out media minus tryptophane, with glycerol (1%), lactate (1%), and nonrepressible media with a trace amount of glucose (0.05%) overnight. Subsequently, galactose (2% final concentration) was used to induce the system for 6 h. PBS 1× with 1% bovine serum albumin was utilized for binding and washing, and readings were taken using a Novocyte Flow Cytometer (Agilent Scientific) as per the manufacturer's instructions.

Antigen: The cDNA of the 1N3R Tau protein isoform7 (NP_001190180.1) with a V5-tag inserted between E73 and A74 was cloned in the pET15b vector, and purification was carried out following the protocol described previously (93). Labeling was realized using Alexa Fluor 488 NHS ester (Thermo Fisher Scientific), with a molecular ratio of 2:1 for NHS Alexa:Tau-V5. Binding analysis was performed using FACS, with serial dilutions of labeled Tau-V5 ranging from 500 nM to 0.98 nM. Data were subjected to nonlinear least-squares regression analysis using the sigmoidal dose-response function provided in GraphPad Prism 9.0. Data presented are representative of one biological replicate (n = 1) in triplicate and represents the mean of % fraction bound.

## Data availability

Crystal structure is available from Protein Data Bank (https://www.rcsb.org/), PDB # 8SKJ.

The data that support the findings of this study are available from the corresponding authors upon reasonable request.

## Supporting information

This article contains supporting information.

## Conflict of interests

The authors declare that they have no conflicts of interests with the contents of this article.

## References

[1] Milligan G., andWhite J.H. (2001). Protein-protein interactions at G-protein-coupled receptors. Trends Pharmacol. Sci..

[2] Seychell B.C., andBeck T. (2021). Molecular basis for protein-protein interactions. Beilstein J. Org. Chem..

[3] Kuzmanov U., andEmili A. (2013). Protein-protein interaction networks: probing disease mechanisms using model systems. Genome Med..

[4] Rao V.S., Srinivas K., Sujini G.N., andKumar G.N. (2014). Protein-protein interaction detection: methods and analysis. Int. J. Proteomics.

[5] Bell M.R., Engleka M.J., Malik A., andStrickler J.E. (2013). To fuse or not to fuse: what is your purpose?. Protein Sci..

[6] Newman R.H., Fosbrink M.D., andZhang J. (2011). Genetically encodable fluorescent biosensors for tracking signaling dynamics in living cells. Chem. Rev..

[7] Villalobos V., Naik S., andPiwnica-Worms D. (2007). Current state of imaging protein-protein interactions *in vivo* with genetically encoded reporters. Annu. Rev. Biomed. Eng..

[8] Guo S., Zhao T., Yun Y., andXie X. (2022). Recent progress in assays for GPCR drug discovery. Am. J. Physiol. Cell Physiol..

[9] Sun Y., Rombola C., Jyothikumar V., andPeriasamy A. (2013). Forster resonance energy transfer microscopy and spectroscopy for localizing protein-protein interactions in living cells. Cytometry A.

[10] Wade M., Mendez J., Coussens N.P., Arkin M.R., Glicksman M.A., Markossian S., Grossman A., Brimacombe K., Arkin M., Auld D., Austin C. (2004). Assay Guidance Manual.

[11] Kimple M.E., Brill A.L., andPasker R.L. (2013). Overview of affinity tags for protein purification. Curr. Protoc. Protein Sci..

[12] Kuey C., Larocque G., Clarke N.I., andRoyle S.J. (2019). Unintended perturbation of protein function using GFP nanobodies in human cells. J. Cell Sci..

[13] Zhao X., Li G., andLiang S. (2013). Several affinity tags commonly used in chromatographic purification. J. Anal. Methods Chem..

[14] Brilhante-da-Silva N., de Oliveira Sousa R.M., Arruda A., Dos Santos E.L., Marinho A.C.M., Stabeli R.G. (2021). Camelid single-domain antibodies for the development of potent diagnosis platforms. Mol. Diagn. Ther..

[15] Dingus J.G., Tang J.C.Y., Amamoto R., Wallick G.K., andCepko C.L. (2022). A general approach for stabilizing nanobodies for intracellular expression. Elife.

[16] Marschall A.L., Dubel S., andBoldicke T. (2015). Specific *in vivo* knockdown of protein function by intrabodies. MAbs.

[17] Bates A., andPower C.A. (2019). David vs. Goliath: the structure, function, and clinical prospects of antibody fragments. Antibodies (Basel).

[18] Hudson P.J., andKortt A.A. (1999). High avidity scFv multimers; diabodies and triabodies. J. Immunol. Methods.

[19] Kabayama H., Takeuchi M., Tokushige N., Muramatsu S.I., Kabayama M., Fukuda M. (2020). An ultra-stable cytoplasmic antibody engineered for in vivo applications. Nat. Commun..

[20] Guglielmi L., Denis V., Vezzio-Vie N., Bec N., Dariavach P., Larroque C. (2011). Selection for intrabody solubility in mammalian cells using GFP fusions. Protein Eng. Des. Sel..

[21] Kvam E., Sierks M.R., Shoemaker C.B., andMesser A. (2010). Physico-chemical determinants of soluble intrabody expression in mammalian cell cytoplasm. Protein Eng. Des. Sel..

[22] Goldman E.R., Liu J.L., Zabetakis D., andAnderson G.P. (2017). Enhancing stability of camelid and shark single domain antibodies: an overview. Front. Immunol..

[23] Flicker S., Zettl I., andTillib S.V. (2020). Nanobodies-useful tools for allergy treatment?. Front. Immunol..

[24] Muyldermans S. (2021). A guide to: generation and design of nanobodies. FEBS J..

[25] Steeland S., Vandenbroucke R.E., andLibert C. (2016). Nanobodies as therapeutics: big opportunities for small antibodies. Drug Discov. Today.

[26] Soetens E., Ballegeer M., andSaelens X. (2020). An inside job: applications of intracellular single domain antibodies. Biomolecules.

[27] de Beer M.A., andGiepmans B.N.G. (2020). Nanobody-based probes for subcellular protein identification and visualization. Front. Cell Neurosci..

[28] Che T., English J., Krumm B.E., Kim K., Pardon E., Olsen R.H.J. (2020). Nanobody-enabled monitoring of kappa opioid receptor states. Nat. Commun..

[29] Galli V., Sebastian R., Moutel S., Ecard J., Perez F., andRoux A. (2017). Uncoupling of dynamin polymerization and GTPase activity revealed by the conformation-specific nanobody dynab. Elife.

[30] Gulati S., Jin H., Masuho I., Orban T., Cai Y., Pardon E. (2018). Targeting G protein-coupled receptor signaling at the G protein level with a selective nanobody inhibitor. Nat. Commun..

[31] Jullien D., Vignard J., Fedor Y., Bery N., Olichon A., Crozatier M. (2016). Chromatibody, a novel non-invasive molecular tool to explore and manipulate chromatin in living cells. J. Cell Sci..

[32] Keller L., Bery N., Tardy C., Ligat L., Favre G., Rabbitts T.H. (2019). Selection and characterization of a nanobody biosensor of GTP-bound RHO activities. Antibodies (Basel).

[33] Kruse A.C., Ring A.M., Manglik A., Hu J., Hu K., Eitel K. (2013). Activation and allosteric modulation of a muscarinic acetylcholine receptor. Nature.

[34] Livingston K.E., Mahoney J.P., Manglik A., Sunahara R.K., andTraynor J.R. (2018). Measuring ligand efficacy at the mu-opioid receptor using a conformational biosensor. Elife.

[35] Morgenstern T.J., Park J., Fan Q.R., andColecraft H.M. (2019). A potent voltage-gated calcium channel inhibitor engineered from a nanobody targeted to auxiliary Ca(V)beta subunits. Elife.

[36] Schenck S., Kunz L., Sahlender D., Pardon E., Geertsma E.R., Savtchouk I. (2017). Generation and characterization of anti-VGLUT nanobodies acting as inhibitors of transport. Biochemistry.

[37] Singh S., Murillo G., Chen D., Parihar A.S., andMehta R.G. (2018). Suppression of breast cancer cell proliferation by selective single-domain antibody for intracellular STAT3. Breast Cancer (Auckl).

[38] Staus D.P., Wingler L.M., Strachan R.T., Rasmussen S.G., Pardon E., Ahn S. (2014). Regulation of beta2-adrenergic receptor function by conformationally selective single-domain intrabodies. Mol. Pharmacol..

[39] Truttmann M.C., Wu Q., Stiegeler S., Duarte J.N., Ingram J., andPloegh H.L. (2015). HypE-specific nanobodies as tools to modulate HypE-mediated target AMPylation. J. Biol. Chem..

[40] Van Impe K., Bethuyne J., Cool S., Impens F., Ruano-Gallego D., De Wever O. (2013). A nanobody targeting the F-actin capping protein CapG restrains breast cancer metastasis. Breast Cancer Res..

[41] Wilton E.E., Opyr M.P., Kailasam S., Kothe R.F., andWieden H.J. (2018). sdAb-DB: the single domain antibody database. ACS Synth. Biol..

[42] De Genst E.J., Guilliams T., Wellens J., O'Day E.M., Waudby C.A., Meehan S. (2010). Structure and properties of a complex of alpha-synuclein and a single-domain camelid antibody. J. Mol. Biol..

[43] Braun M.B., Traenkle B., Koch P.A., Emele F., Weiss F., Poetz O. (2016). Peptides in headlock--a novel high-affinity and versatile peptide-binding nanobody for proteomics and microscopy. Sci. Rep..

[44] Virant D., Traenkle B., Maier J., Kaiser P.D., Bodenhofer M., Schmees C. (2018). A peptide tag-specific nanobody enables high-quality labeling for dSTORM imaging. Nat. Commun..

[45] Cabalteja C.C., Sachdev S., andCheloha R.W. (2022). Characterization of a nanobody-epitope tag interaction and its application for receptor engineering. ACS Chem. Biol..

[46] Boersma S., Khuperkar D., Verhagen B.M.P., Sonneveld S., Grimm J.B., Lavis L.D. (2019). Multi-color single-molecule imaging uncovers extensive heterogeneity in mRNA decoding. Cell.

[47] Lutje Hulsik D., Liu Y.Y., Strokappe N.M., Battella S., El Khattabi M., McCoy L.E. (2013). A gp41 MPER-specific llama VHH requires a hydrophobic CDR3 for neutralization but not for antigen recognition. PLoS Pathog..

[48] Jin-jing LI F.X., Yan-wei J.I., Mei S.H.U., Zhui T.U., Jin-heng F.U. (2018). Biopanning of anti c-Myc-tag nanobodies and its application for bioimaging China. Biotechnology.

[49] Traenkle B., Emele F., Anton R., Poetz O., Haeussler R.S., Maier J. (2015). Monitoring interactions and dynamics of endogenous beta-catenin with intracellular nanobodies in living cells. Mol. Cell Proteomics.

[50] Cheloha R.W., Harmand T.J., Wijne C., Schwartz T.U., andPloegh H.L. (2020). Exploring cellular biochemistry with nanobodies. J. Biol. Chem..

[51] Gotzke H., Kilisch M., Martinez-Carranza M., Sograte-Idrissi S., Rajavel A., Schlichthaerle T. (2019). The ALFA-tag is a highly versatile tool for nanobody-based bioscience applications. Nat. Commun..

[52] Vigano M.A., Ell C.M., Kustermann M.M.M., Aguilar G., Matsuda S., Zhao N. (2021). Protein manipulation using single copies of short peptide tags in cultured cells and in Drosophila melanogaster. Development.

[53] Yang X., Boehm J.S., Yang X., Salehi-Ashtiani K., Hao T., Shen Y. (2011). A public genome-scale lentiviral expression library of human ORFs. Nat. Methods.

[54] Sarov M., Barz C., Jambor H., Hein M.Y., Schmied C., Suchold D. (2016). A genome-wide resource for the analysis of protein localisation in Drosophila. Elife.

[55] Hanke T., andRandall R.E. (1995). Variable domain sequences of mAb with high affinity for a linear oligopeptide. Immunogenetics.

[56] Hanke T., Szawlowski P., andRandall R.E. (1992). Construction of solid matrix-antibody-antigen complexes containing simian immunodeficiency virus p27 using tag-specific monoclonal antibody and tag-linked antigen. J. Gen. Virol..

[57] Randall R.E., Young D.F., Goswami K.K., andRussell W.C. (1987). Isolation and characterization of monoclonal antibodies to simian virus 5 and their use in revealing antigenic differences between human, canine and simian isolates. J. Gen. Virol..

[58] Bartel P.L., andFields S. (1995). Analyzing protein-protein interactions using two-hybrid system. Methods Enzymol..

[59] Fromont-Racine M., Rain J.C., andLegrain P. (1997). Toward a functional analysis of the yeast genome through exhaustive two-hybrid screens. Nat. Genet..

[60] Moutel S., Bery N., Bernard V., Keller L., Lemesre E., de Marco A. (2016). NaLi-H1: a universal synthetic library of humanized nanobodies providing highly functional antibodies and intrabodies. Elife.

[61] Padlan E.A. (1994). Anatomy of the antibody molecule. Mol. Immunol..

[62] Miura N., Miyamoto K., Ohtani Y., Yaginuma K., Aburaya S., Kitagawa Y. (2019). Domain swapping of complementarity-determining region in nanobodies produced by Pichia pastoris. AMB Express.

[63] Mitchell L.S., andColwell L.J. (2018). Analysis of nanobody paratopes reveals greater diversity than classical antibodies. Protein Eng. Des. Sel..

[64] Chao G., Lau W.L., Hackel B.J., Sazinsky S.L., Lippow S.M., andWittrup K.D. (2006). Isolating and engineering human antibodies using yeast surface display. Nat. Protoc..

[65] Danis C., Dupre E., Zejneli O., Caillierez R., Arrial A., Begard S. (2022). Inhibition of Tau seeding by targeting Tau nucleation core within neurons with a single domain antibody fragment. Mol. Ther..

[66] Kroeze W.K., Sassano M.F., Huang X.P., Lansu K., McCorvy J.D., Giguere P.M. (2015). PRESTO-Tango as an open-source resource for interrogation of the druggable human GPCRome. Nat. Struct. Mol. Biol..

[67] Laroche G., andGiguere P.M. (2019). Measurement of beta-arrestin recruitment at GPCRs using the Tango assay. Methods Mol. Biol..

[68] Zeghal M., Laroche G., andGiguere P.M. (2020). Parallel interrogation of beta-arrestin2 recruitment for ligand screening on a GPCR-wide scale using PRESTO-tango assay. J. Vis. Exp..

[69] Kim M.W., Wang W., Sanchez M.I., Coukos R., von Zastrow M., andTing A.Y. (2017). Time-gated detection of protein-protein interactions with transcriptional readout. Elife.

[70] Zeghal M., Laroche G., Freitas J.D., Wang R., andGiguere P.M. (2023). Profiling of basal and ligand-dependent GPCR activities by means of a polyvalent cell-based high-throughput platform. Nat. Commun..

[71] Sun S., Yang X., Wang Y., andShen X. (2016). *In Vivo* analysis of protein-protein interactions with bioluminescence resonance energy transfer (BRET): progress and prospects. Int. J. Mol. Sci..

[72] Loening A.M., Fenn T.D., Wu A.M., andGambhir S.S. (2006). Consensus guided mutagenesis of Renilla luciferase yields enhanced stability and light output. Protein Eng. Des. Sel..

[73] Bertrand L., Parent S., Caron M., Legault M., Joly E., Angers S. (2002). The BRET2/arrestin assay in stable recombinant cells: a platform to screen for compounds that interact with G protein-coupled receptors (GPCRS). J. Recept Signal. Transduct. Res..

[74] Jensen A.A., Hansen J.L., Sheikh S.P., andBrauner-Osborne H. (2002). Probing intermolecular protein-protein interactions in the calcium-sensing receptor homodimer using bioluminescence resonance energy transfer (BRET). Eur. J. Biochem..

[75] Dixon A.S., Schwinn M.K., Hall M.P., Zimmerman K., Otto P., Lubben T.H. (2016). NanoLuc complementation reporter optimized for accurate measurement of protein interactions in cells. ACS Chem. Biol..

[76] Nickolls S.A., Humphreys S., Clark M., andMcMurray G. (2013). Co-expression of GRK2 reveals a novel conformational state of the micro-opioid receptor. PLoS One.

[77] Wan Q., Okashah N., Inoue A., Nehme R., Carpenter B., Tate C.G. (2018). Mini G protein probes for active G protein-coupled receptors (GPCRs) in live cells. J. Biol. Chem..

[78] Culhane K.J., Gupte T.M., Madhugiri I., Gadgil C.J., andSivaramakrishnan S. (2022). Kinetic model of GPCR-G protein interactions reveals allokairic modulation of signaling. Nat. Commun..

[79] Wagner T.R., andRothbauer U. (2020). Nanobodies right in the middle: intrabodies as toolbox to visualize and modulate antigens in the living cell. Biomolecules.

[80] Stadler C., Rexhepaj E., Singan V.R., Murphy R.F., Pepperkok R., Uhlen M. (2013). Immunofluorescence and fluorescent-protein tagging show high correlation for protein localization in mammalian cells. Nat. Methods.

[81] Ruffolo J.A., Chu L.S., Mahajan S.P., andGray J.J. (2023). Fast, accurate antibody structure prediction from deep learning on massive set of natural antibodies. Nat. Commun..

[82] McMahon C., Baier A.S., Pascolutti R., Wegrecki M., Zheng S., Ong J.X. (2018). Yeast surface display platform for rapid discovery of conformationally selective nanobodies. Nat. Struct. Mol. Biol..

[83] Lobstein J., Emrich C.A., Jeans C., Faulkner M., Riggs P., andBerkmen M. (2012). SHuffle, a novel Escherichia coli protein expression strain capable of correctly folding disulfide bonded proteins in its cytoplasm. Microb. Cell Fact.

[84] Rabia L.A., Desai A.A., Jhajj H.S., andTessier P.M. (2018). Understanding and overcoming trade-offs between antibody affinity, specificity, stability and solubility. Biochem. Eng. J..

[85] Pathan H., andWilliams J. (2012). Basic opioid pharmacology: an update. Br. J. Pain.

[86] Dasgupta C., andZhang L. (2011). Angiotensin II receptors and drug discovery in cardiovascular disease. Drug Discov. Today.

[87] Salahudeen M.S., andNishtala P.S. (2017). An overview of pharmacodynamic modelling, ligand-binding approach and its application in clinical practice. Saudi Pharm. J..

[88] Milligan G. (1999). Exploring the dynamics of regulation of G protein-coupled receptors using green fluorescent protein. Br. J. Pharmacol..

[89] El Khamlichi C., Reverchon-Assadi F., Hervouet-Coste N., Blot L., Reiter E., andMorisset-Lopez S. (2019). Bioluminescence resonance energy transfer as a method to study protein-protein interactions: application to G protein coupled receptor biology. Molecules.

[90] Wouters E., Vasudevan L., Crans R.A.J., Saini D.K., andStove C.P. (2019). Luminescence- and fluorescence-based complementation assays to screen for GPCR oligomerization: current state art. Int. J. Mol. Sci..

[91] Chen X., Zaro J.L., andShen W.C. (2013). Fusion protein linkers: property, design and functionality. Adv. Drug Deliv. Rev..

[92] Vojtek A.B., andHollenberg S.M. (1995). Ras-Raf interaction: two-hybrid analysis. Methods Enzymol..

[93] Danis C., Despres C., Bessa L.M., Malki I., Merzougui H., Huvent I. (2016). Nuclear magnetic resonance spectroscopy for the identification of multiple phosphorylations of intrinsically disordered proteins. J. Vis. Exp..

