# Supplementary material for: Development of a V5-tag–directed nanobody and its implementation as an intracellular biosensor of GPCR signaling
Source: J Biol Chem. 2023 Jul 28;299(9):105107. doi: 10.1016/j.jbc.2023.105107 (PMC10470007; doi:10.1016/j.jbc.2023.105107)
Supplement: Supporting information 1 [file mmc1.pptx]

## Slide 1
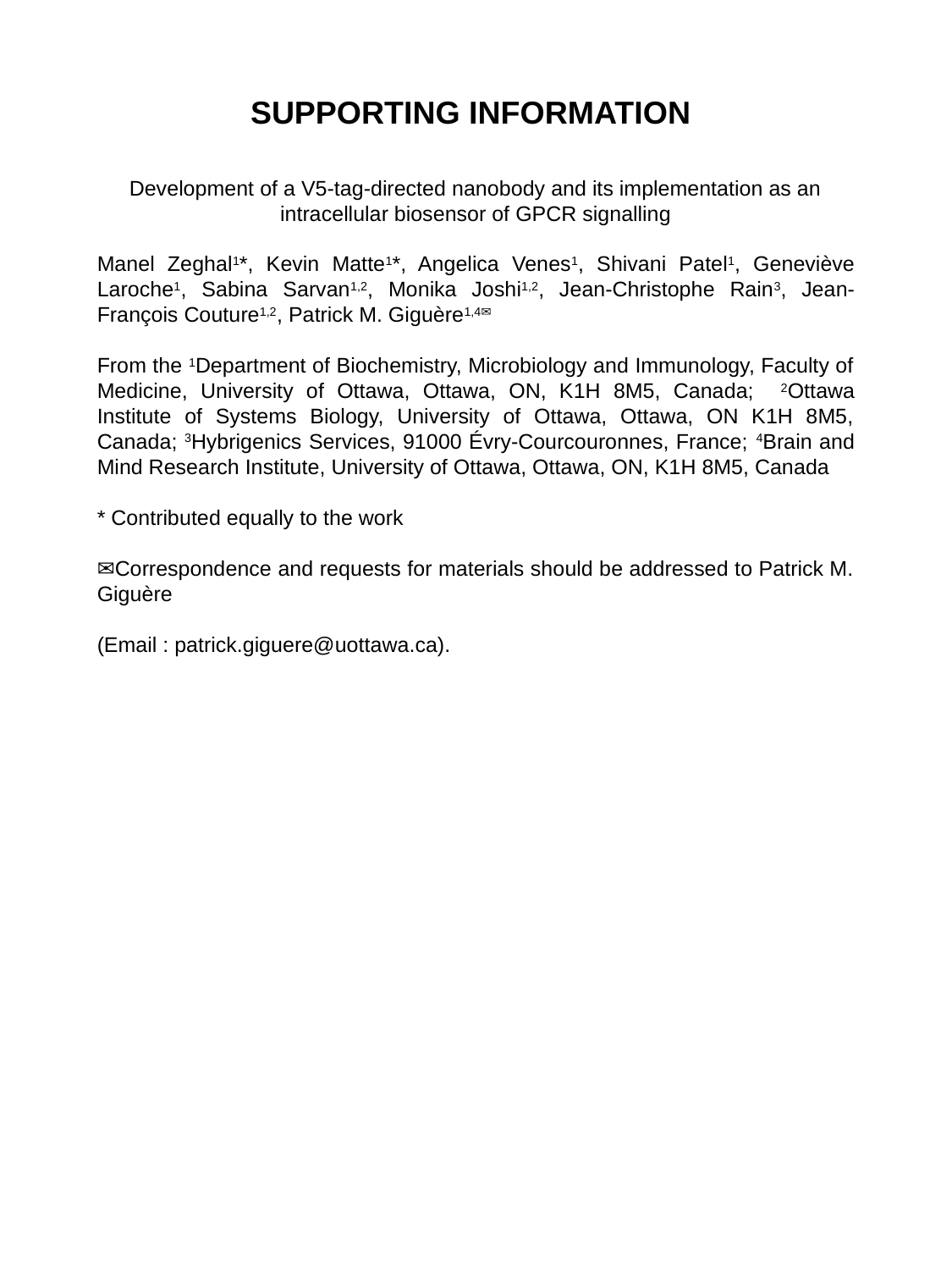

SUPPORTING INFORMATION
Development of a V5-tag-directed nanobody and its implementation as an intracellular biosensor of GPCR signalling
Manel Zeghal1*, Kevin Matte1*, Angelica Venes1, Shivani Patel1, Geneviève Laroche1, Sabina Sarvan1,2, Monika Joshi1,2, Jean-Christophe Rain3, Jean-François Couture1,2, Patrick M. Giguère1,4✉️
From the 1Department of Biochemistry, Microbiology and Immunology, Faculty of Medicine, University of Ottawa, Ottawa, ON, K1H 8M5, Canada; 2Ottawa Institute of Systems Biology, University of Ottawa, Ottawa, ON K1H 8M5, Canada; 3Hybrigenics Services, 91000 Évry-Courcouronnes, France; 4Brain and Mind Research Institute, University of Ottawa, Ottawa, ON, K1H 8M5, Canada
* Contributed equally to the work
✉️Correspondence and requests for materials should be addressed to Patrick M. Giguère
(Email : patrick.giguere@uottawa.ca).

## Slide 2
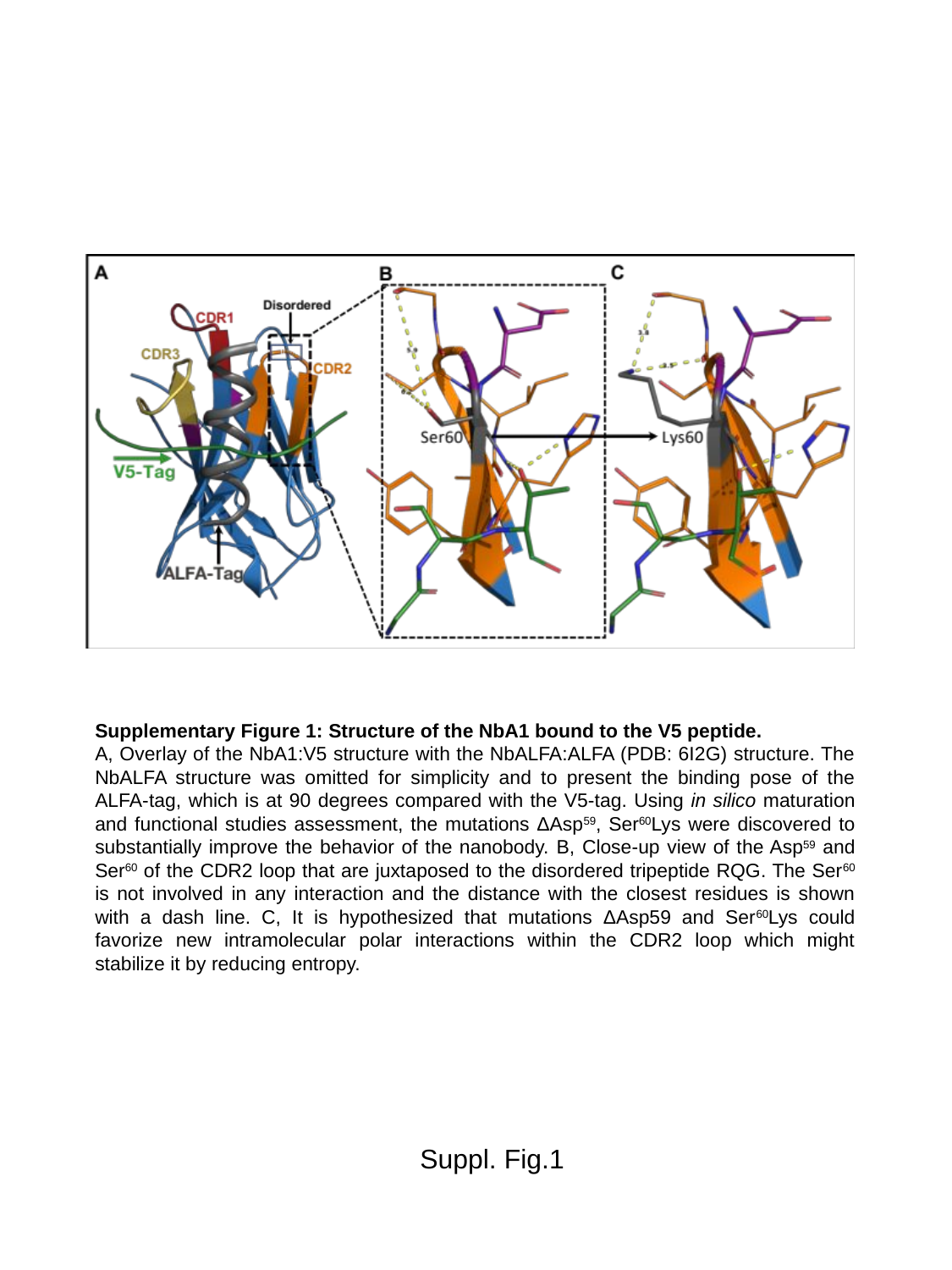

Supplementary Figure 1: Structure of the NbA1 bound to the V5 peptide.
A, Overlay of the NbA1:V5 structure with the NbALFA:ALFA (PDB: 6I2G) structure. The NbALFA structure was omitted for simplicity and to present the binding pose of the ALFA-tag, which is at 90 degrees compared with the V5-tag. Using in silico maturation and functional studies assessment, the mutations ΔAsp59, Ser60Lys were discovered to substantially improve the behavior of the nanobody. B, Close-up view of the Asp59 and Ser60 of the CDR2 loop that are juxtaposed to the disordered tripeptide RQG. The Ser60 is not involved in any interaction and the distance with the closest residues is shown with a dash line. C, It is hypothesized that mutations ΔAsp59 and Ser60Lys could favorize new intramolecular polar interactions within the CDR2 loop which might stabilize it by reducing entropy.
Suppl. Fig.1

## Slide 3
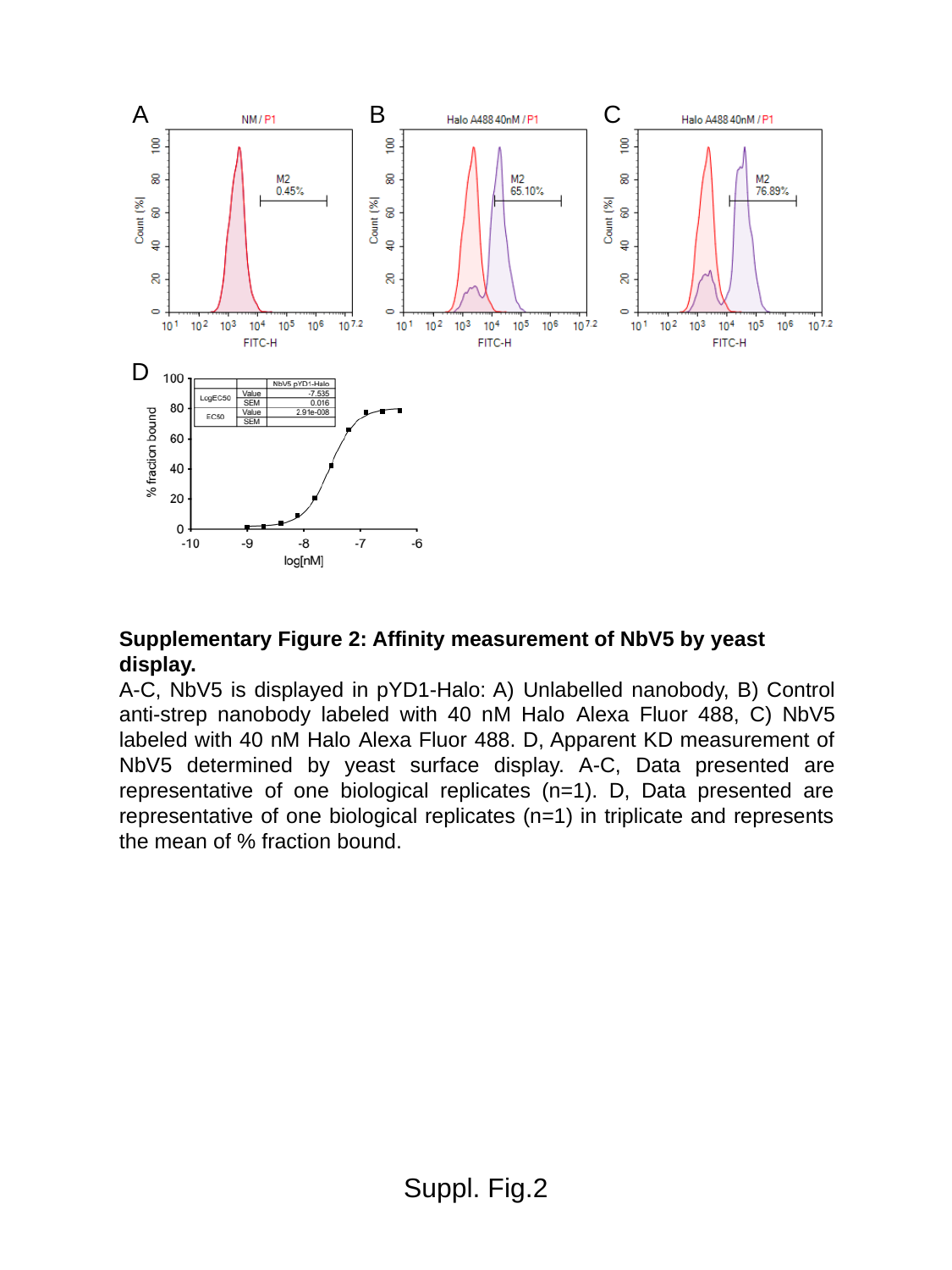

A
B
C
D
Supplementary Figure 2: Affinity measurement of NbV5 by yeast display.
A-C, NbV5 is displayed in pYD1-Halo: A) Unlabelled nanobody, B) Control anti-strep nanobody labeled with 40 nM Halo Alexa Fluor 488, C) NbV5 labeled with 40 nM Halo Alexa Fluor 488. D, Apparent KD measurement of NbV5 determined by yeast surface display. A-C, Data presented are representative of one biological replicates (n=1). D, Data presented are representative of one biological replicates (n=1) in triplicate and represents the mean of % fraction bound.
Suppl. Fig.2

## Slide 4
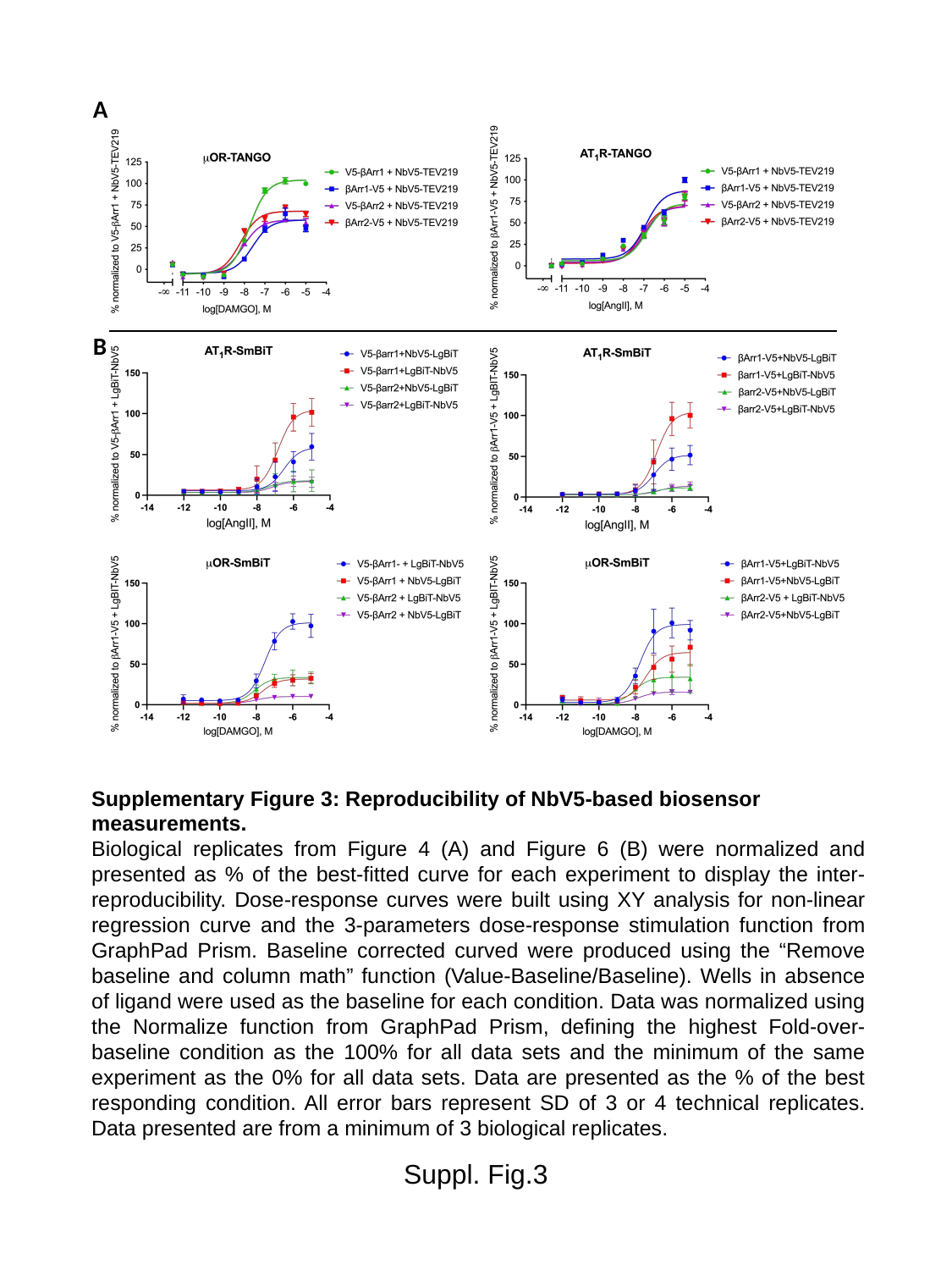

A
B
Supplementary Figure 3: Reproducibility of NbV5-based biosensor measurements.
Biological replicates from Figure 4 (A) and Figure 6 (B) were normalized and presented as % of the best-fitted curve for each experiment to display the inter-reproducibility. Dose-response curves were built using XY analysis for non-linear regression curve and the 3-parameters dose-response stimulation function from GraphPad Prism. Baseline corrected curved were produced using the “Remove baseline and column math” function (Value-Baseline/Baseline). Wells in absence of ligand were used as the baseline for each condition. Data was normalized using the Normalize function from GraphPad Prism, defining the highest Fold-over-baseline condition as the 100% for all data sets and the minimum of the same experiment as the 0% for all data sets. Data are presented as the % of the best responding condition. All error bars represent SD of 3 or 4 technical replicates. Data presented are from a minimum of 3 biological replicates.
Suppl. Fig.3

## Slide 5
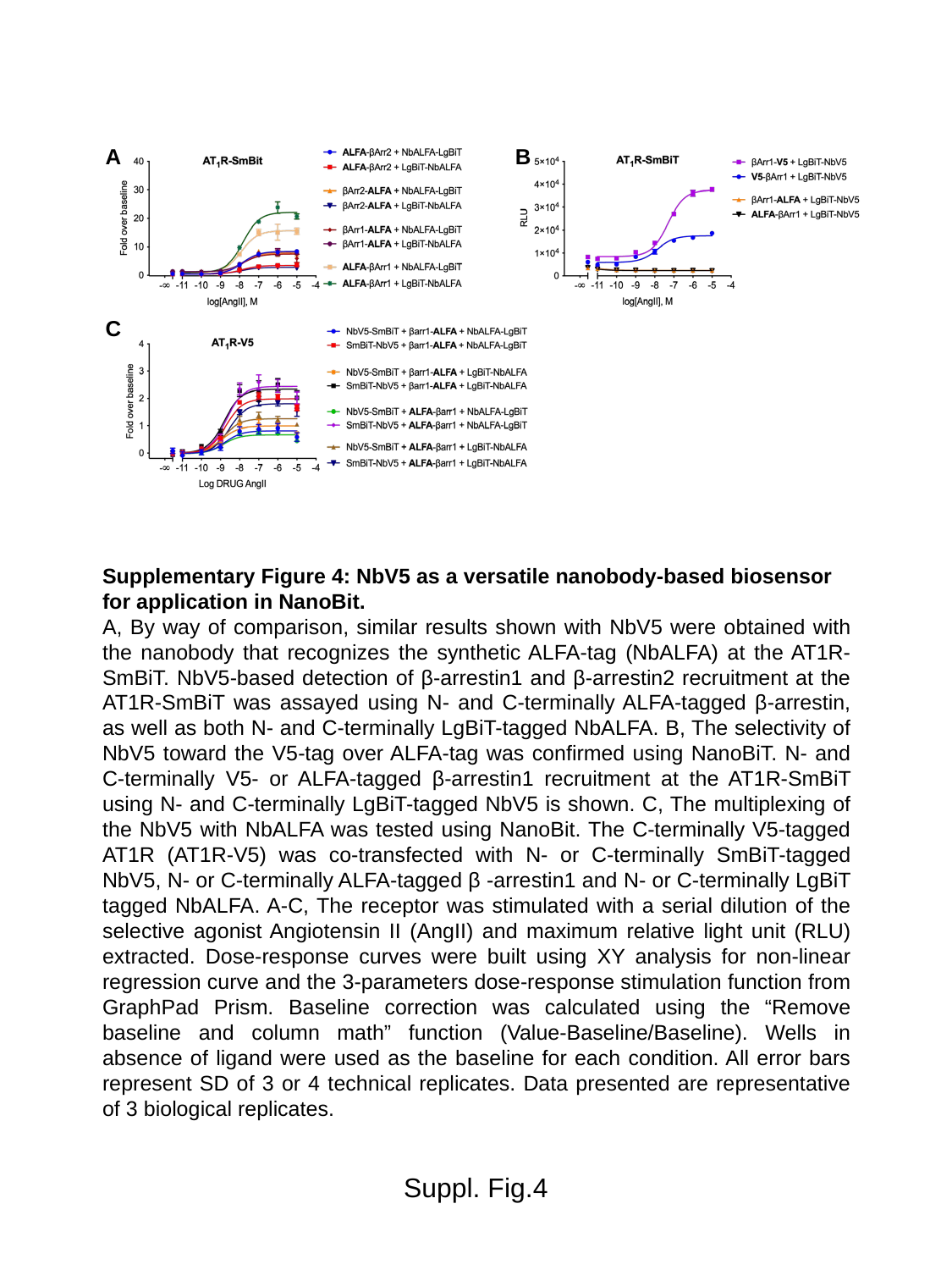

A
B
C
Supplementary Figure 4: NbV5 as a versatile nanobody-based biosensor for application in NanoBit.
A, By way of comparison, similar results shown with NbV5 were obtained with the nanobody that recognizes the synthetic ALFA-tag (NbALFA) at the AT1R-SmBiT. NbV5-based detection of β-arrestin1 and β-arrestin2 recruitment at the AT1R-SmBiT was assayed using N- and C-terminally ALFA-tagged β-arrestin, as well as both N- and C-terminally LgBiT-tagged NbALFA. B, The selectivity of NbV5 toward the V5-tag over ALFA-tag was confirmed using NanoBiT. N- and C-terminally V5- or ALFA-tagged β-arrestin1 recruitment at the AT1R-SmBiT using N- and C-terminally LgBiT-tagged NbV5 is shown. C, The multiplexing of the NbV5 with NbALFA was tested using NanoBit. The C-terminally V5-tagged AT1R (AT1R-V5) was co-transfected with N- or C-terminally SmBiT-tagged NbV5, N- or C-terminally ALFA-tagged β -arrestin1 and N- or C-terminally LgBiT tagged NbALFA. A-C, The receptor was stimulated with a serial dilution of the selective agonist Angiotensin II (AngII) and maximum relative light unit (RLU) extracted. Dose-response curves were built using XY analysis for non-linear regression curve and the 3-parameters dose-response stimulation function from GraphPad Prism. Baseline correction was calculated using the “Remove baseline and column math” function (Value-Baseline/Baseline). Wells in absence of ligand were used as the baseline for each condition. All error bars represent SD of 3 or 4 technical replicates. Data presented are representative of 3 biological replicates.
Suppl. Fig.4
